# New Functions of Mitochondrial Dysfunction in Gastric Cancer: From Molecular Processes to Potential Treatments

**DOI:** 10.3390/ijms27146305

**Published:** 2026-07-15

**Authors:** Huanhuan Liu, Yating Zhang, Juan Wang, Min Qiao, Qinghong Guo

**Affiliations:** 1 The First School of Clinical Medicine, Lanzhou University, Lanzhou 730000, China; lhh3499379861@163.com (H.L.);; 2 Department of Gastroenterology, Gansu Province Clinical Research Center for Digestive Diseases, The First Hospital of Lanzhou University, Lanzhou 730000, China

**Keywords:** gastric cancer, mitochondrial dysfunction, metabolic reprogramming, mitochondrial dynamics, tumor microenvironment, targeted therapy

## Abstract

Through multifaceted reprogramming, mitochondria, the fundamental organelles of eukaryotic cells, can drive the malignant growth of malignancies. They also control energy metabolism, redox balance, and cell fate determination. Due to its high heterogeneity and primary/acquired drug resistance, gastric cancer (GC), a highly deadly and common cancer worldwide, continues to present significant clinical treatment challenges. Current targeted and immunotherapy strategies have been unable to significantly improve long-term patient survival. Thus, the pathological roles and molecular mechanisms of mitochondrial dysfunction (including mutations in mitochondrial DNA, imbalances in mitochondrial dynamics, aberrant mitophagy, abnormalities in mitochondrial permeability transition pores, and metabolic disorders) in the development of GC are systematically reviewed in this article. The specific mitochondrial phenotypic remodeling of various molecular subtypes of GC, abnormalities in membrane contact interactions between mitochondria and other organelles, the regulatory roles of mitochondrial dysfunction in tumor microenvironment (TME) immune evasion, maintenance of tumor stemness, and ferroptosis, as well as their major effects on the malignant progression and treatment resistance of GC, are all thoroughly examined. In order to provide important theoretical references and novel research perspectives for identifying therapeutic targets with greater precision, clarifying resistance mechanisms, and developing novel combination strategies in GC, this article summarizes the current research status and translational potential of anti-GC therapeutic strategies targeting mitochondria. It also explores the translational challenges currently faced in this field and the core future research directions.

## 1. Introduction

According to 2024 global cancer statistics, GC continues to be the fourth most prevalent cause of cancer-related mortality and the fifth most common malignant tumor, accounting for over 650,000 fatalities and approximately 1 million new cases annually [1,2]. The global disease burden of GC is predicted to rise by 62% by 2040, despite the fact that its incidence is decreasing [3]. The four molecular subtypes of GC identified by The Cancer Genome Atlas (TCGA) are microsatellite instability (MSI), chromosomal instability (CIN), genomically stable (GS), and Epstein–Barr virus-associated (EBV) [4,5]. Tumor diagnosis and treatment have changed from traditional cytotoxic chemotherapy to safer and more effective molecular-targeted therapy as a result of the advancement of high-throughput sequencing and multi-omics technology. Treatment approaches for solid tumors have also been altered. Nevertheless, even though sequencing research has provided a thorough understanding of the molecular traits and essential mechanisms of GC [6], due to problems like drug resistance and limited applicable populations, current strategies like chemotherapy, targeted therapy (such as anti-HER2 therapy), and immune checkpoint inhibitors have not significantly improved overall survival; the 5-year overall survival rate for all stage GC patients is approximately 25%, and the 5-year survival rate for advanced metastatic patients remains below 5% [7]. As the primary center of cellular energy metabolism and homeostatic regulation, mitochondria have a crucial role in the development, progression, and resistance to treatment of GC that has to be thoroughly investigated.

Although mitochondrial dysfunction has been widely implicated in cancer biology, its role in GC should not be understood merely as a collection of isolated abnormalities in energy metabolism, oxidative stress, or apoptosis. In order to clarify the entire pathological process of mitochondrial dysfunction, from the beginning of precancerous lesions to the development of terminal malignant phenotypes, this paper proposes a four-level damage cascade amplification model driven by mitochondrial dysfunction based on available research evidence. At the first level, carcinogenic stimuli and microenvironmental stresses, particularly H. pylori infection, chronic inflammation, hypoxia, and nutrient deprivation, induce mitochondrial genomic injury and oxidative phosphorylation impairment, leading to mtDNA mutations or deletions, respiratory chain dysfunction, excessive reactive oxygen species production, and disturbed ATP homeostasis [8]. At the second level, persistent mitochondrial stress disrupts mitochondrial quality control (QC), resulting in imbalanced fission–fusion dynamics, aberrant mitophagy, and dysregulated mitochondrial permeability transition pore opening [9]. At the third level, failure of mitochondrial QC drives metabolic and phenotypic reprogramming, including altered glucose, lipid, and amino acid metabolism, apoptotic escape, ferroptosis resistance, and maintenance of cancer stem cell-like properties [10,11,12]. At the fourth level, mitochondrial dysfunction extends beyond tumor cells to remodel the tumor microenvironment (TME) through metabolic competition, mtDNA leakage, organelle-level communication, and immune–metabolic crosstalk, thereby promoting immune evasion and resistance to chemotherapy, targeted therapy, and immunotherapy [13,14]. The entire malignant evolution of GC, from precancerous lesions to carcinoma in situ, invasion, metastasis, and treatment resistance, is driven by the four levels, which gradually amplify one another and offer mutual feedback.

Based on this framework, this review systematically elucidates the multifaceted regulatory roles of mitochondrial dysfunction in GC from the perspectives of mtDNA mutations, mitochondrial quality control (QC), mitochondrial permeability transition pore abnormalities, metabolic reprogramming, and the interactions between mitochondrial dysfunction and the TME, cancer stem cells, and ferroptosis, with a particular focus on the underlying mechanisms and therapeutic intervention strategies. In addition, this review comprehensively summarizes the current research progress in mitochondria-targeted anti-GC therapies, discusses the major challenges hindering their clinical translation, and outlines key future research directions. By doing so, this review aims to address the gaps in existing studies regarding inter-organelle communication, molecular subtype-based stratification, and immune–metabolic crosstalk in GC.

## 2. The Mechanism and Process of Regulating Mitochondrial Homeostasis

In eukaryotic cells, mitochondria are essential organelles for signal transduction, energy metabolism, and cell fate determination. A fundamental requirement for preserving the stability of the cellular internal environment is mitochondrial homeostasis, which is defined as the dynamic equilibrium in their abundance, structure, function, and genomic integrity [15]. Four main functioning modules must be coordinated in order to maintain this balance. The first is material and energy metabolism. Mitochondria are the primary location for the TCA cycle, fatty acid β-oxidation, and amino acid catabolism, which finish the crucial intermediate stages of protein, lipid, and carbohydrate metabolism [16]. Secon is the oxidative phosphorylation system: through electron transfer, the respiratory chain complexes in the inner membrane of the mitochondria create a transmembrane electrochemical gradient that propels the synthesis of adenosine triphosphate (ATP) and supplies the primary energy for cellular life activities [17]. The fourth is the management of cell fate, which preserves population homeostasis by eliminating cells with seriously damaged mitochondria; the third is the mitochondrial QC system, which preserves its structural and functional integrity through fission, fusion, and mitophagy [18]. Furthermore, the homeostasis of ions like calcium, iron, and copper within cells is mostly regulated by mitochondria. Together with apoptosis, abnormalities in ion homeostasis can cause new types of programmed cell death, including ferroptosis and cuproptosis, creating a comprehensive network that allows mitochondria to control cell fate [19].

Leigh syndrome, mitochondrial myopathy–encephalopathy–lactic acidosis–stroke-like episodes syndrome, and chronic progressive external ophthalmoplegia are examples of primary mitochondrial diseases, which are primarily caused by defects in mtDNA replication, maintenance, and translation. Secondary mitochondrial dysfunction results from the pathological progression of several prevalent disorders, including metabolic syndrome, heart failure, and neurodegenerative diseases [20]. All pathways intersect and are precisely coupled to maintain normal mitochondrial function under pathological conditions. These pathways include proteins and genomic QC at the molecular level, dynamics, autophagy, and biogenesis at the organelle level, and organelle interactions and nucleus–mitochondria signal communication at the cellular level [21].

## 3. GC and Mitochondrial Dysfunction

**Figure 1:**
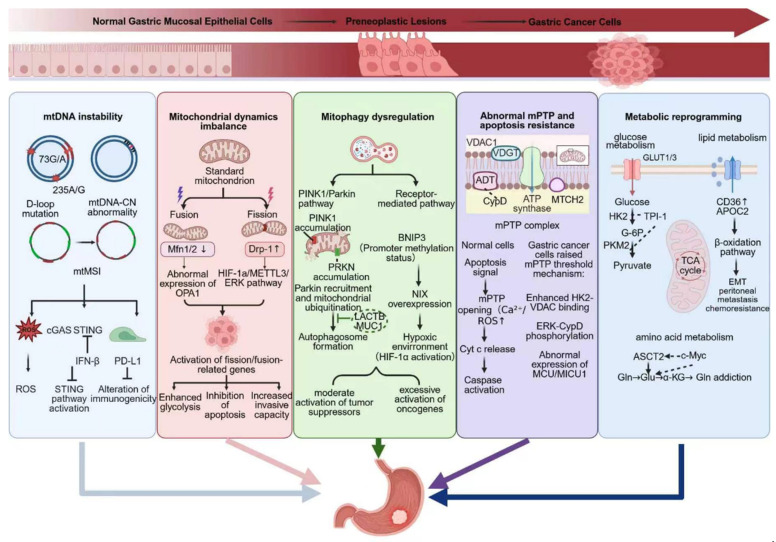
Mechanisms by which mitochondrial dysfunction drives gastric cancer progression. Mitochondrial dysfunction promotes the transition from normal gastric mucosa to gastric cancer through mtDNA instability, imbalance of mitochondrial dynamics, dysregulated mitophagy, abnormal mPTP opening, and metabolic reprogramming. These alterations induce ROS accumulation, apoptosis resistance, immune evasion, enhanced glycolysis, lipid and amino acid metabolic remodeling, and activation of invasion- and metastasis-related pathways, ultimately facilitating gastric cancer initiation, progression, and chemoresistance. Arrows indicate pathological progression, mechanistic regulation, metabolic flow, or convergence of mitochondrial dysfunction modules. T-shaped lines denote inhibitory effects.

Maintaining normal stomach function depends on mitochondrial integrity, and an imbalance in homeostasis or signal transduction can seriously impair mucosal health and hasten the course of disease. Stomach pit cells, parietal cells, chief cells, and neuroendocrine cells make up the majority of the stomach mucosa; parietal cells are the primary functional cells that secrete gastric acid [22]. Due to their extremely high metabolic demands, parietal cells rely heavily on mitochondrial function. In addition to providing the large amounts of ATP needed by the H^+^/K^+^-ATPase proton pump for gastric acid secretion, mitochondria also play a role in controlling calcium signals associated with acid secretion and gastric motility. Additionally, the tight junctions that preserve the integrity of the gastric mucosal barrier are highly dependent on mitochondrial energy production [23]. One of the main causes of GC is persistent inflammation brought on by chronic damage, and mitochondria are essential in this process by controlling signaling pathways like NF-κB [24]. Numerous investigations have shown distinctive mitochondrial anomalies in gastric lesions: parietal cells show malfunctioning “giant mitochondria” in starving models [25], molecular alterations include aberrant mitochondrial localization of the MUC1 protein, downregulation of the tumor suppressor protein genes associated with retinoid-interferon-induced mortality 19, activation of the NOXO1/ROS signaling pathway, and damage to mitochondrial DNA in human precancerous gastric lesions [26,27,28,29]. With an emphasis on investigating targeted therapeutic strategies against important nodes in this process, this section will methodically clarify the fundamental pathogenic mechanisms of mitochondrial dysfunction in GC in accordance with the hierarchical logic of the previously mentioned cascade model. The goal is to provide a foundation for further understanding the pathogenesis of GC, as well as for the development of novel diagnostic and therapeutic approaches (Figure 1).

### 3.1. MtDNA Instability

The mutation rate of mtDNA is 10–100 times higher than that of nuclear DNA, and it lacks histone protection, has low mismatch repair effectiveness, and is found at the central locations of mitochondrial ROS production [30,31]. It is prone to copy number decrease or sequence alterations, which can lead to a range of illnesses, including cancer. Point mutations and enrichment of single-nucleotide polymorphisms, abnormal copy number (mtDNA-CN), and mitochondrial microsatellite instability (mtMSI) are the primary manifestations of mtDNA instability in GC. These factors together cause mitochondrial dysfunction and the malignant phenotype of GC. High-frequency allele variations in the D-loop region, including 73G/A, 235A/G, 309C/C insertion, 324C/G, 16362T/C, and 16519C/T, are significantly linked to an increased risk of GC, while the 523–524AC/del variation lowers the risk of disease, according to case–control studies. This suggests that single-nucleotide polymorphisms in the D-loop region could be used as potential molecular markers for determining GC risk [32].

Using a case–control study design, Zhu Xun and colleagues discovered that peripheral blood leukocyte mtCN levels were considerably higher in Chinese patients with GC than in the control group (median: 6.53 vs. 4.12). Independent of telomere length, the incidence of GC increased by 29% and 74% in the medium and high mtCN groups, respectively, as compared to the low mtCN group, suggesting promise as a biomarker [33]. High expression of MYO15A (*p* = 0.036), OSGEP (*p* = 0.008), and CDK10 (*p* = 0.036) were all significantly linked to lower overall survival in patients with GC, according to Yang Qizhou and colleagues, who also discovered that an increase in peripheral blood mtDNA-CN in GC showed a significant causal effect on shorter overall survival (HR = 72.97, 95% CI: 68.86–77.08, *p* = 0.046) [34]. However, genetically predicted mtDNA-CN was not significantly causally associated with the risk of 20 cancers, including GC (OR = 1.02, 95% CI: 0.95–1.10), according to a Mendelian randomization analysis based on large cohorts like the UK Biobank, which does not support its use for tumor risk assessment [35]. Intra-tumoral mtDNA heterogeneity, sample type, tumor stage, molecular subtype, and ethnic variations could all contribute to this result’s variability. Therefore, large multicenter cohorts are still required to validate the predictive significance of mtDNA-CN in GC. Microsatellite sequences like polycytidine and dinucleotide repeats are abundant in mtDNA, and aberrant repeat lengths make up mtMSI. Research has demonstrated that mtMSI is a common genetic event in GC, particularly enriched in the D-loop hotspot region; mtMSI positivity is strongly linked to distal gastric tumors, intestinal-type GC, and later stages of TNM. It may contribute to the development of GC and is associated with *H. pylori* infection, inflammation, and nuclear genome instability [36].

By causing ROS accumulation, mitochondrial membrane remodeling, and STING pathway activation, mtDNA instability in GC enhances anti-tumor immunity; isoflavonoid targets DHODH to generate mitochondrial damage, which can further activate the STING pathway to exert an immune-stimulating impact [37]. It should be made clear that the majority of current research supports a strong link between aberrant mtDNA alterations and the occurrence, development, proliferation, invasion, and metastasis of GC. It is still debatable, nevertheless, whether mtDNA mutations are the primary cause of GC or if they only occur as the tumor progresses. Furthermore, there is still a lack of study on the clonal selection of mtDNA mutations and the co-evolution of nuclear genomes and mitochondria in the development of GC, which is a crucial area for further investigation.

### 3.2. Unbalanced Mitochondrial Dynamics: Fission and Fusion Dysregulation

The primary mechanism for preserving mitochondrial shape and functional balance is mitochondrial dynamics. It takes part in important physiological processes such as energy metabolism, redox homeostasis, and apoptosis through the dynamic balance of fission (controlled by DRP1 and Mff) and fusion (regulated by MFN1/2 and OPA1) [38,39,40]. Hypoxia, acidosis, and oxidative stress can upset this equilibrium in the TME, causing an imbalance in mitochondrial dynamics and serving as a crucial mechanism for tumor cells to adapt to the milieu and gain a growth advantage [41]. Abnormalities in mitochondrial dynamics are frequently seen in GC, and they usually show up as excessive fission and insufficient fusion, which causes the mitochondrial network to split [42]. Karthik Balakrishnan and colleagues discovered that this disorder pattern displays subtype heterogeneity: intestinal-type GC is enriched with signals for mitochondrial biosynthesis and renewal, while diffuse-type GC has significantly activated gene sets related to mitochondrial fission, fusion, and localization, which is closely linked to its high migration and metastatic characteristics [43]. The aforementioned findings show that an imbalance in mitochondrial dynamics is a major element actively promoting the malignant transformation and evolution of GC rather than a passive concurrent event.

The most typical dynamic abnormality in GC is excessive stimulation of mitochondrial fission. Its primary regulatory factor, “dynamin-related protein 1 (DRP1, encoded by the Dynamin 1 Like Gene),” is a dependable prognostic marker since it is markedly overexpressed in GC tissues and is independently linked to tumor infiltration depth, lymph node metastasis, and poor prognosis [44,45,46]. DRP1 activation in GC is controlled in several ways: a hypoxic microenvironment increases the m^2^ at the post-translational modification level. DRP1 is modified via the HIF-1α/METTL3 axis to stabilize its protein [47]. Through phosphorylation of the DRP1 Ser616 site (ERK-DRP1 axis), research on the natural substance Sanggenon C has, for the first time, demonstrated the critical function of the ERK signaling pathway [48]. In terms of epigenetic regulation, the m6A demethylase Fat Mass and Obesity-Associated Protein can indirectly disrupt mitochondrial dynamic homeostasis through modulation of downstream signaling molecules [49,50]. According to other research, indomethacin can activate protein kinase C, which increases mitochondrial fission and causes p38 phosphorylation and DRP1 activation. This finally causes both normal and GC cells to undergo apoptosis [51]. Severe mitochondrial fragmentation brought on by excessive DRP1 activation not only interferes with oxidative phosphorylation and produces a spike in mitochondrial reactive oxygen species (mtROS), but it also serves as a foundation for the growth, survival, invasion, and metastasis of cancer cells by encouraging glycolysis and blocking apoptotic pathways.

The majority of research indicates that “mitofusin 2 (MFN2)” expression is downregulated in GC tissues as compared to normal gastric mucosa, and that overexpressing MFN2 in vitro can stop the growth of GC cells and cause apoptosis [52]. However, the contradictory results of the Chia-LangFang team show that MFN2 expression is higher in GC tumor tissues than in normal tissues and that MFN2 silencing can stop cell growth [53]. The functional heterogeneity may result from variations in tumor molecular subtypes. In order to understand the functional differences of MFN2 in various tumor cell subpopulations, it will be important in the future to create specialized antibodies that can identify different modification states and subcellular localization of MFN2 in conjunction with single-cell sequencing technologies.

### 3.3. Mitophagy’s Two-Sided Sword Role

Through the autophagy–lysosome pathway, mitophagy serves as the primary mechanism for breaking down damaged mitochondria and preserving mitochondrial homeostasis [54,55]. Numerous studies have demonstrated that mitophagy has a “double-edged sword” effect in tumors: it suppresses tumor growth or cell carcinogenesis in the early stages of tumor development, but in metastatic tumors that have already developed, it gives cells a survival advantage and prevents chemotherapy-induced cell death [56,57]. Simultaneously, its aberrant enhancement or functional weakening might worsen genomic instability, accumulate damaged mitochondria and oxidative stress, and ultimately encourage the growth of tumors [58,59]. Therefore, the condition and subtype of cancer cells mostly determine whether mitophagy has pro-cancer or antitumor effects.

Parkin RBR E3 ubiquitin ligase (PRKN) and PTEN-induced kinase 1 (PINK1) are essential molecules that control mitophagy and preserve mitochondrial quality [60,61]. While there are deletions or loss-of-function mutations in the PRKN gene in a number of cancers, including glioblastoma, ovarian cancer, and breast cancer, there is increased mitophagy activity in esophageal squamous cell carcinoma and lung cancer tissues [62]. The PINK1/Parkin pathway is frequently aberrantly activated in GC and is strongly associated with chemotherapy resistance and tumor cell survival [63]. By upregulating PINK1, Parkin, and the LC3B-II/I ratio, metformin markedly increased mitophagy in GC cells and reduced ATP production, shielding cancer cells from cisplatin-induced cytotoxicity and promoting treatment resistance, according to an in vitro study [64]. By blocking PINK1/Parkin-mediated mitophagy in AGS GC cell lines, overexpression of LACTB can preserve mitochondrial homeostasis and lower ROS generation, preventing cell death and accelerating tumor growth [65]. Through the MUC1/ATAD3A/PINK1 axis, the oncoprotein MUC1 can increase cancer cell proliferation, stem cell properties, and tumorigenic potential in vivo. These pro-cancer effects can be considerably reversed by blocking MUC1 or mitophagy [66].

In addition to the PINK1/Parkin pathway, receptor-mediated mitophagy is essential for GC’s adaptability to a hypoxic microenvironment. The outer mitochondrial membrane receptor proteins BNIP3 (BCL2/adenovirus E1B19kDa interacting protein 3) and NIX (sometimes called BNIP3L) can bind directly to the autophagosome marker protein LC3, facilitating hypoxia-induced mitophagy [58,62]. BNIP3 is frequently suppressed in GC tissues as a result of promoter hypermethylation [67,68]. BNIP3 is poorly expressed in GC, according to bioinformatics analysis, and miRNA-214-3p can control autophagy and hasten the malignant development of GC by targeting BNIP3 [69]. By causing demethylation of the BNIP3 promoter and downregulating the expression of the long-chain non-coding RNA PVT1, methionine deprivation can stop the growth of GC cells by triggering mitophagy [70]. Furthermore, Hirofumi Sugita and associates verified that the objective response rate to chemotherapy was only 16.7% in patients with BNIP3 promoter methylation in a cohort of advanced GC patients receiving fluoropyrimidine plus platinum-based chemotherapy. This was significantly lower than the 52.8% in patients without methylation (*p* = 0.003). Additionally, the methylated group had significantly lower median progression-free survival (2.8 months vs. 5.1 months) and median overall survival (8.3 months vs. 13.6 months), indicating that BNIP3 methylation is a reliable indicator of poor chemotherapy response and a poor prognosis [71]. According to recent studies, succinate can enhance anti-tumor immunity and improve clinical outcomes in GC immunotherapy by activating BNIP3-mediated mitophagy, which preserves the metabolic health and stem-like properties of CD8^+^ T cells [72]. The aforementioned findings imply that BNIP3 has a tumor-suppressive impact in particular circumstances. Unlike BNIP3, NIX is linked to a poor prognosis and is increased in GC. NIX deficiency significantly prolongs survival and suppresses tumor growth in tumor-bearing mouse models.

### 3.4. Resistance to Cell Death and Abnormal Mitochondrial Permeability Transition Pore (mPTP)

mPTP is a key mitochondrial node linking calcium overload and oxidative stress to apoptosis. Transient mPTP opening helps maintain mitochondrial homeostasis, whereas sustained opening under strong stress causes mitochondrial membrane potential collapse, cytochrome c release, and caspase activation [73,74]. In GC, mPTP-related apoptosis resistance may involve several mechanisms. First, mitochondrial carrier homolog 2 (MTCH2), an upstream regulator associated with mPTP, is highly expressed in GC and promotes malignant phenotypes by regulating mitochondrial function, membrane potential, ATP production, ATP2A2 expression, and mPTP opening [75]. Second, dysregulated mitochondrial calcium handling may increase apoptotic resistance and platinum resistance. High MCU expression is associated with adverse clinicopathological features, altered mitochondrial metabolism, ROS production, apoptosis regulation, and platinum resistance, suggesting a link between calcium signaling, mPTP control, and therapeutic response [76]. Third, mitochondrial damage does not necessarily induce cell death in GC. Sublethal mitochondrial stress can activate protective retrograde signaling, such as the ROS-GCN2-eIF2α-ATF4-xCT axis, thereby enhancing antioxidant capacity and promoting cisplatin resistance [73]. Thus, mPTP-related mitochondrial stress has dual effects in GC: severe pore opening promotes apoptosis, whereas adaptive mitochondrial stress supports survival and chemoresistance.

Preclinical investigations have verified the significance of mPTP in the development and response to treatment of GC. By triggering Cyclophilin D(CypD)-dependent mPTP opening, the naturally occurring coumarin compound esculetin can cause human SGC-7901, MGC-803, and BGC-823 GC cells to undergo apoptosis. Mechanistically, esculetin appears to act at the mitochondrial death-execution level by increasing ROS-associated CypD-dependent mPTP opening, leading to mitochondrial membrane permeabilization and apoptosis. However, esculetin is not GC-specific, and the current evidence remains limited to preclinical GC cell models [77]. Collectively, mPTP-related mechanisms in GC may regulate apoptotic threshold, metabolic adaptation, invasion, and chemotherapy response. However, most available agents are not GC-specific, and direct clinical evidence remains limited. Future studies should validate these mechanisms in subtype-stratified models, patient-derived organoids, and in vivo systems, while developing biomarkers such as MTCH2, MCU, CypD activity, mitochondrial calcium load, and membrane potential to guide precise targeting of mPTP-related vulnerabilities.

In addition to mPTP-dependent mitochondrial permeability regulation, resistance to cell death in GC is also closely associated with an increased mitochondrial apoptotic threshold governed by the BCL-2 family. Anti-apoptotic members such as BCL-2, BCL-XL, and MCL-1 maintain mitochondrial integrity by restraining BH3-only proteins and the effector proteins BAX and BAK, whereas pro-apoptotic members promote mitochondrial outer membrane permeabilization, cytochrome c release, and caspase activation [78]. In GC, this balance is often shifted toward cell survival. Increased BCL-2 expression and MCL-1-mediated protection against 5-FU- or cisplatin-induced apoptosis suggest that anti-apoptotic buffering is an important mitochondrial adaptation contributing to treatment resistance [79,80].

Because GC cells may depend on different anti-apoptotic BCL-2 family members, BCL-2 family profiling is important for identifying apoptosis-related vulnerabilities. Current evidence suggests that BCL-XL and MCL-1, rather than BCL-2 alone, may represent relevant survival dependencies in selected GC models [81,82]. Therefore, assessment of BCL-2, BCL-XL, MCL-1, BAX, BAK, and key BH3-only proteins may help stratify tumors according to their apoptotic dependency and guide the selection of rational combination strategies. BH3 profiling provides a functional method to evaluate mitochondrial apoptotic priming. By measuring mitochondrial responses to different BH3 peptides, this approach can infer dependence on specific anti-apoptotic proteins and predict sensitivity to apoptosis-inducing therapies [83]. In GC, BH3 profiling has been associated with docetaxel sensitivity, and BAK expression has been linked to treatment response and survival in patients receiving docetaxel-containing preoperative chemotherapy [84]. Thus, BH3 profiling may be useful for treatment stratification in locally advanced GC before perioperative therapy and in advanced or refractory GC when selecting individualized therapeutic combinations.

### 3.5. Disorder of Mitochondrial Metabolism

The reprogramming of the mitochondrial metabolic network, which serves as the central hub linking mitochondrial structural damage with malignant cellular phenotypes, is a common downstream functional outcome of the previously mentioned mitochondrial genome damage, dynamic imbalance, autophagy abnormalities, and abnormal mPTP opening [10]. One of the main causes of GC is metabolic dysregulation, which can cause cells to undergo substantial metabolic reprogramming to sustain quick cell division and survival in stressful situations [16]. Apart from the traditional Warburg effect, amino acid and lipid metabolism are also profoundly altered. Different pathways work in concert to control energy supply, material synthesis, and signal transduction, which impacts GC proliferation, immune evasion, and treatment resistance [85].

#### 3.5.1. Disorder of Glucose Metabolism

Aerobic glycolysis is a fundamental metabolic characteristic of gastric solid tumors, and glycolytic flow is markedly increased in GC [86,87]. The glucose transporter (GLUT) family is primarily responsible for mediating glucose uptake: by modulating glucose uptake, GLUT3 might contribute to macrophage M2 polarization in the TME and is a possible biomarker for GC prognosis and immune infiltration. It is also strongly linked to greater TNM stage and poor survival [88,89]. For instance, Hans Anton Schlücker and associates examined specimens from 150 patients with GC who underwent total gastrectomy. They discovered that the mean overall survival of GLUT3-positive patients was only 38.6 months, which was significantly shorter than the 51.2 months of GLUT3-negative patients (*p* = 0.028), and that the risk of death was 85.2% higher for GLUT3-positive patients (HR = 1.852) [90]. The rate-limiting enzyme in the first stage of glycolysis is hexokinase 2 (HK2), which catalyzes the phosphorylation of glucose to produce glucose-6-phosphate (G-6P) [91]. According to recent research, triosephosphate isomerase-1 can interact with HK2; knocking down triosephosphate isomerase-1 can significantly downregulate the expression of HK2 and PKM2, inhibiting the proliferation and invasion of GC cells; and mechanistically, triosephosphate isomerase-1 drives glycolytic reprogramming by persistently activating the first steps of glycolysis [92]. Additionally, a poor prognosis in GC is indicated by the important glycolytic enzyme enolase 1 (ENO1). According to survival studies, GC patients with ENO1 overexpression had a lower overall survival rate, and MGC-803 and MKN-45 GC cells’ capacity to proliferate and form colonies can be inhibited by ENO1 knockdown [93]. By increasing GLUT3 expression and stimulating lactate production, this hypoxic enzyme can improve cellular glucose uptake and metabolism. This ultimately drives the stem cell characteristics of GC, the expression of markers related to the epithelial–mesenchymal transition (EMT), self-renewal, migration, and invasion [94]. As a crucial enzyme in the last stage of glycolysis, PKM2 catalyzes the transformation of phosphoenolpyruvate into pyruvate and ATP. The dynamic equilibrium between dimeric and tetrameric PKM2 is upset in GC. Dimeric PKM2 can directly decrease the activity of mitochondrial electron transport chain complexes, decreasing the efficiency of oxidative phosphorylation, and translocate to the nucleus to function as a transcriptional co-activator controlling gene expression [95]. Clinical applications of the basic pathways of metabolic reprogramming in GC have steadily emerged. ^1^F-fluorodeoxyglucose PET-CT was able to identify metastases missed by traditional scans in 7% of patients, of which approximately 5% would not be discovered if just conventional staging was conducted, according to a study comprising 279 patients with GC without metastasis according to conventional CT staging [96].

#### 3.5.2. Disorder of Lipid Metabolism

Increased fatty acid absorption, elevated fatty acid oxidation, and aberrant cholesterol metabolism are all signs of lipid metabolic reprogramming, a fundamental aspect of mitochondrial dysfunction in GC. Tumor cells obtain ATP and reducing equivalents from mitochondrial fatty acid β-oxidation, but chemoresistance and metastasis are tightly linked to cholesterol metabolic remodeling [95]. Lipid and triglyceride levels are typically higher in GC; metabolomics analysis reveals that 3-hydroxybutyrate, the final product of fatty acid β-oxidation in GC cells, is reduced, cholesterol synthesis is inhibited, and the rat GC model also shows abnormal blockage of fatty acid degradation, oxidative stress, and amino acid metabolism pathways [85]. The fatty acid translocase CD36 and carnitine palmitoyltransferase 1A (CPT1A) are currently the fatty acid metabolism enzymes implicated with GC. After being activated by acyl-CoA synthetase, long-chain fatty acids can either enter mitochondria for β-oxidation or be esterified into triglycerides and stored in lipid droplets. In the event of hypoxia or glucose deprivation, free fatty acids released from lipid droplets provide energy through oxidation to support tumor cell survival [97]. In GC, CD36 is overexpressed and promotes fatty acid uptake by working in concert with APOC2 to activate the PI3K-AKT-mTOR axis, which causes an epithelial–mesenchymal transition. Meanwhile, CD36-mediated lipid peroxidation and ferroptosis can suppress CD8^+^ tumor-infiltrating lymphocyte activity, which ultimately results in immune evasion and peritoneal metastasis [98,99]. Long-chain fatty acids are transported into the mitochondrial matrix by the rate-limiting fatty acid oxidation enzyme CPT1A. PD-1^+^ T cells in the TME can upregulate CPT1A expression, resulting in increased lipolysis and fatty acid oxidation while suppressing glycolysis and amino acid metabolism. CPT1A expression is upregulated in GC and is closely linked to the maintenance of tumor stemness and chemotherapy resistance. These findings suggest that CPT1A may act as a key mediator linking lipid metabolism to immune regulation in GC [100].

Circular RNA circUBR5 is markedly elevated in gastric signet ring cell carcinoma (GSRCC), according to recent research, and its high expression is linked to advanced malignancies, metastasis, and a poor prognosis [101]. Cytoplasmic circUBR5 drives the reprogramming of cholesterol metabolism through two mechanisms: first, it functions as a molecular sponge for miR-1208, relieving its inhibition on CYP19A1 and initiating the estrogen signaling pathway; second, it binds directly to the cholesterol esterifying enzyme ACAT1 and recruits the deubiquitinase PSMD14 to stabilize its expression. Furthermore, circUBR5 can disseminate chemoresistance in the TME through exosomes; cisplatin and antisense oligonucleotides that target circUBR5 can work in concert to stop tumor development and reverse resistance. This discovery offers a possible treatment target for the highly invasive GSRCC subtype by revealing a novel mechanism by which non-coding RNAs control cholesterol metabolism to promote GC development [101].

#### 3.5.3. Reprogramming Amino Acid Metabolism: Mitochondrial Substrate Supply and Glutamine Catabolism

In GC, glutamine metabolism participates in mitochondrial substrate replenishment and supports malignant progression partly by regulating tumor-cell survival and immune responses; tryptophan metabolism may contribute to immunosuppression through the kynurenine-related signaling axis and cytokine-mediated pathways; and arginine metabolism can modulate the functional states of immune cells within the tumor microenvironment [102]. After entering the cell via the transporter ASCT2, glutamine is processed by glutaminase (GLS) in the mitochondria to produce glutamate, which is subsequently transformed into α-ketoglutarate by either transaminase or glutamate dehydrogenase to enter the TCA cycle [103]. By transcriptionally stimulating ASCT2 and GLS expression, the c-Myc oncoprotein plays a crucial regulatory role in this process, causing the “Gln addiction” phenotype [104]. Glutamine competition between tumors and immune cells may impact anti-tumor immunity, as c-Myc also upregulates glutamine metabolism genes during early T cell activation [105]. In vitro and in vivo experiments have demonstrated that the combined inhibition of ASCT2 and GS can effectively block the glutamine pathway in cancer cells, providing a new potential target for the treatment of GC [106]. Furthermore, GLS1 expression in GC cells can be increased by the buildup of lactic acid in an acidic environment, which encourages glutamine catabolism [107]. Tumor-associated macrophages express transglutaminase 2 (TGM2), which can stimulate the NF-κB and ERK1/2 pathways, causing the release of IL-1β, CSF-1, and MMPs. This promotes the growth and metastasis of GC cells [108,109]. By activating STAT1 and preventing its ubiquitin-mediated degradation, TGM2 can also increase pro-tumor effects, according to research by Zhang and colleagues [110]. By binding to TGM2, the targeting peptide GX1 can diminish VEGF expression, block the NF-κB/HIF1α pathway, restrict its GTP-binding activity, and slow the progression of GC [111]. Transcriptome sequencing and metabolic analysis revealed that the long-chain non-coding RNA NR_033928 drives glutamine metabolism reprogramming, increases the production of glutamate and α-ketoglutarate, and upregulates GLS expression to promote GC cell proliferation and inhibit apoptosis both in vivo and in vitro [112]. Using a large TCGA cohort, Li Weidong and colleagues developed a prognostic signature based on glutamine metabolism genes, demonstrating that the low-risk group’s survival was substantially higher than that of the high-risk group and that it accurately predicted the prognosis of GC patients [113].

#### 3.5.4. GC Metabolic Reprogramming and *H. pylori* Infection

Through a variety of virulence factors, *H. pylori* directly controls the metabolic reprogramming of GC cells, causing mitochondrial dysfunction and malignant transformation. As a key oncogenic protein, CagA activates the NF-κB pathway to upregulate PKM2 and PDK1 expression. It also localizes to the mitochondria to limit SIRT3 deacetylase activity, stabilizing HIF-1α and improving the glycolytic phenotype [114]. Furthermore, CagA can cause the epithelial–mesenchymal transition via the PI3K-AKT-mTOR axis and increase the expression of lipid translocase CD36 to enhance the uptake of free fatty acids [115]. In addition to causing increased mitochondrial fission, decreased mtDNA copy number, and loss of membrane potential, which results in mitochondrial dysfunction, VacA toxin increases lipid influx by creating membrane channels [116]. *H. pylori* enhances glutamine catabolism at the level of amino acid metabolism by upregulating the expression of ASCT2 and GLS1, which replenishes carbon sources for the TCA cycle. At the same time, it induces IDO1 activity, which promotes regulatory T cell differentiation through the tryptophan–kynurenine–aromatic hydrocarbon receptor axis, creating an immunosuppressive microenvironment [117]. *H. pylori* inhibits the TCA Cycle’s activity in the early stages of infection to start glycolytic conversion; as the infection worsens, the cycle is altered to produce biosynthetic precursors [118,119]. The central role of mitochondrial stress in the development of GC is confirmed by in vitro experiments that demonstrate that the mitochondrial stress response protein lon protease 1 is induced early in infection and maintains basic mitochondrial function by activating the mitochondrial unfolded protein response; knocking down lon protease 1 can reverse *H. pylori*-induced metabolic changes [120]. Lipopolysaccharide stimulates macrophage Arg1 expression to encourage M2 polarization, upregulates PDK1 to prevent glycolysis, and activates NK cell NF-κB signaling through TLR4 [95,121]. In conclusion, *H. pylori* targets mitochondrial metabolism, modifies the glucose–lipid–amino acid metabolic network, and creates an immunosuppressive milieu, among other multi-level pathways that drive the development of GC.

#### 3.5.5. Metabolic and Phenotypic Reprogramming in Various GC Subtypes

The CIN subtype is characterized by a high frequency of TP53 mutations [122]. A prominent metabolic feature of this subtype is highly active glycolysis. Mutant p53 loses its transcriptional repression of GLUT1 and HK2, thereby driving enhanced glucose uptake and glycolytic activity [123,124]. Simultaneously, EGFR/MET amplification enhances the binding of HK2 to mitochondrial VDAC, preventing acid-induced apoptosis, and activates the PI3K-AKT-mTORC1 axis, further upregulating glycolytic genes by stabilizing HIF-1α [125]. A metabolism-epigenetic regulatory positive feedback loop is formed when highly expressed PKM2 undergoes nuclear translocation, forms a complex with HIF-1α and JMJD3, and relieves the epigenetic repression of glycolytic genes through H3K27me3 demethylation [126,127]. This ultimately creates a hypoxic microenvironment marked by lactic acid accumulation and T cell exhaustion.

The MSI subtype is characterized by a high mutational burden resulting from defects in DNA mismatch repair, with amino acid dependence representing a central metabolic feature. IFN-γ constantly activates the tryptophan–kynurenine axis by upregulating IDO1 through the JAK-STAT1 pathway, which results in local tryptophan depletion and suppression of effector T cell activity [128,129]. This subtype of glutamine metabolism is very adaptive: when resources are adequate, ASCT2 is increased to improve exogenous glutamine utilization; under food scarcity, the AMPK/STAT3 pathway upregulates glutamine synthetase to sustain the TCA cycle by endogenous synthesis [130].

Viral-mediated epigenetic silencing via metabolites is the fundamental characteristic of the EBV-positive subtype [131]. Pro-cancer metabolites, including fumarate and D-2-hydroxyglutarate, build up as a result of EBV latent infection’s upregulation of ASCT2 and GLS1 to improve glutamine catabolism [132,133]. By acting as competitive inhibitors of α-ketoglutarate, these metabolites cause extensive chromatin condensation and a CpG island methylator phenotype (CIMP) by blocking the activity of TET DNA demethylases and KDM histone demethylases [134,135]. Furthermore, excessive promoter methylation silences the genes encoding MHCI molecules, making it difficult for tumor cells to effectively convey viral antigens to CD8 T cells, which leads to immune evasion [136].

The crux of the GS subtype is morphological remodeling rather than conventional metabolic reprogramming, and it is typified by mutations in CDH1 and RHOA, which correspond to the diffuse type in Lauren’s classification. Actin cytoskeleton rearrangement is driven by the RHOA-ROCK axis, which increases cell motility [137]; TGF-β signaling creates a positive feedback loop with this axis, preserving the mesenchymal phenotype [138,139]. It is primarily distinguished metabolically by somewhat elevated fatty acid oxidation [140], which supplies energy and raw materials for membrane biosynthesis and cytoskeletal remodeling, ultimately resulting in early peritoneal metastasis and a poor prognosis [141].

In conclusion, each GC molecular subtype has unique metabolic reprogramming traits: GS facilitates morphological remodeling by fatty acid oxidation, EBV enables epigenetic silencing through metabolites, MSI depends on amino acid metabolism, and CIN is focused on glycolysis. These subtype-specific metabolic characteristics offer a theoretical foundation for immunotherapy and targeted therapy.

### 3.6. Immune Evasion, Mitochondria, and the TME

Through a variety of mechanisms, including metabolite secretion, mtDNA leakage, and organelle-level transfer, mitochondrial dysfunction within tumor cells not only determines the malignant phenotype of the cells themselves but can also spill over into the extracellular microenvironment, changing the metabolic state and functional phenotype of immune cells and ultimately promoting tumor immune evasion [13]. Tumor immune evasion, anti-tumor immunological responses, immune cell function, and dispersion are all influenced by the quantity and quality of mitochondria. T cell persistence can be improved by increased mitochondrial biomass and spare respiratory capacity, giving them a bioenergetic advantage to kill cancer cells and stop recurrence [142,143]. Furthermore, mitochondria control the direction of immune cell migration, facilitating their recruitment to tumor locations and moving toward regions with high chemokine concentrations [144]. Immune cell survival and function can be preserved by low levels of mitochondrial reactive oxygen species (mROS), particularly by boosting T cell receptor (TCR) signaling upon tumor antigen stimulation [145]. The prognostic model based on mitochondria-related genes (MTRGs) can accurately predict the effectiveness of immunotherapy and is closely linked to the infiltration levels of different immune cells in the TME, including B cells, regulatory T cells (Tregs), and tumor-infiltrating T lymphocytes (TILs) [146]. In conclusion, immune cell antitumor effects can be directly weakened by mitochondrial dysfunction, which can also encourage tumor immune evasion.

#### 3.6.1. In the GC Microenvironment, Mitochondria Have an Impact on Immune Cell Fatigue and Metabolic Competition

One of the main characteristics of the TME is the battle between tumor cells and invading immune cells for food metabolism. TILs experience glucose deprivation as a result of tumor cells’ competitive uptake of glucose from the microenvironment through aerobic glycolysis [147]. Excessive mROS produced by dysfunctional mitochondria in a hypoxic environment sustains the activation of activated T cell nuclear factor (NFAT), upregulates genes associated with fatigue mediated by TOX and NR4A, and finally causes CD8^+^ T cell exhaustion [148]. OXPHOS-related genes (NDUFB8, SDHB) were downregulated, oxidative stress indicators (GPX1, SOD2) were increased, and IFN-γ and granzyme B secretion were decreased in exhausted CD8^+^ T cells, according to a transcriptome study of TILs in GC patients [149,150]. Furthermore, peroxisome proliferator-activated receptor gamma coactivator 1-alpha (PGC-1α) can be phosphorylated by chronic activation of the PI3K-AKT pathway in GC TILs, which inhibits its transcriptional activity and impairs mitochondrial biogenesis, aggravating T cell metabolic paralysis [151]. High NK cell infiltration is linked to improved neoadjuvant chemotherapy response and prolonged overall life in GC patients, according to clinical investigations by Masanori Oshi and others [152]. However, through the HIF-1α/mTOR-DRP1 pathway, the hypoxic milieu of GC can upregulate DRP1 and downregulate MFN2, causing mitochondrial fragmentation in NK cells, which inhibits granule exocytosis and IFN-γ production and decreases NK cell cytotoxicity [153]. Tumor-associated macrophages (TAMs) are mostly polarized to the M2 phenotype in the GC microenvironment, and mitochondrial metabolism shifts toward OXPHOS and fatty acid oxidation (FAO) [154]. M2-type TAMs suppress T cell activation by secreting TGF-β and IL-10, and their mitochondria-derived succinate builds up in the microenvironment, which may further encourage the migration of GC cells [155]. In conclusion, by controlling the mitochondrial metabolism of different immune cell subsets and establishing an immunosuppressive milieu, GC cells facilitate tumor immune escape.

#### 3.6.2. The Immunogenicity of GC Cells Is Controlled by Mitochondria

How well the immune system recognizes GC cells depends on their immunogenicity. Unusual mtDNA abundance in the cytoplasm can result from mitochondrial dysfunction. As a damage-associated molecular pattern, oxidatively damaged mtDNA (ox-mtDNA) can activate the cGAS-STING pathway, increase the secretion of type I interferons (IFN-α/β), and boost tumor immunogenicity. However, prolonged activation of this pathway can also induce PD-L1 expression, attract myeloid-derived suppressor cells (MDSCs), and cause T cell exhaustion, all of which can promote tumor immune evasion [13]. In gastrointestinal cancer cells, aberrant DRP1 overexpression can cause severe mitochondrial fragmentation and ongoing mtDNA damage [156]. In addition to failing to generate an efficient interferon-mediated anti-tumor response, this chronic low-level mtDNA leaking causes persistent aberrant activation of the cGAS-STING pathway, which also establishes deep immunological tolerance, destroys immune-stimulating proteins, and initiates pro-tumor autophagy [157].

#### 3.6.3. In GC, Mitochondria Control Immunological Response and Antigen Presentation

The effectiveness of GC’s antigen presentation is determined by mitochondrial metabolism, which also controls T cell-mediated anti-tumor immune responses [145]. The mitochondrial division inhibitor Mdivi-1 can upregulate tumor cell surface MHC-I expression, increasing the effectiveness of adoptive T cell therapy. Proteomics and functional studies have confirmed that increased mitochondrial metabolic activity can significantly increase MHC-I expression levels in GC cells [158]. By phosphorylating STAT1, ROS buildup brought on by mitochondrial failure can trigger the MAPK/ERK pathway, which inhibits MHC-I transcription and surface expression [158]. High expression of genes related to mitochondria was shown to be substantially correlated with elevated MHC-I levels and increased CD8^+^ T cell infiltration in GC tissues [159]. Furthermore, studies on mitochondrial transplantation have demonstrated that adding mitochondria from healthy gastric epithelial cells to GC cells might enhance their metabolic performance and indirectly alter the tumor immunological microenvironment [160]. Differential gene enrichment analysis further confirmed that cell adhesion molecules, chemokine signaling, and complement cascade pathways are significantly enriched in the high-risk group, indicating that disruption of mitochondrial homeostasis directly affects tumor antigen presentation efficiency and immune recognition processes. The prognostic model based on mitophagy-related lncRNAs demonstrated that GC patients with high-risk scores have a distinct immune cell infiltration pattern and are closely associated with the activation of B cell receptor and chemokine signaling pathways [161].

#### 3.6.4. Immune Checkpoint Expression in GC Is Influenced by Mitochondria

Immune checkpoint expression and mitochondrial dysfunction in TILs of GC are regulated in both directions. The elevation of PD-1 and Lymphocyte-Activation Gene 3 expression in the GC microenvironment is positively connected with the decreased mitochondrial quality and spare respiratory capacity of CD8^+^ T cells [162]. By upregulating CPT1A, increasing FAO, and blocking glycolysis, PD-1 signaling reprograms T cell metabolism and causes T cell fatigue [100]. CTLA-4 signaling solely impedes T cell effector development by blocking glycolysis, which reduces their antitumor efficacy; it has no effect on FAO [162]. Furthermore, PD-1 activation lowers the number of mitochondrial cristae in T cells, which further diminishes OXPHOS efficiency and prevents memory T cell development [163]. GC immune cells’ mitochondrial function can be adversely regulated by immunological checkpoint molecules. Increased mitochondrial fission and lower membrane potential are linked to TIM-3 expression on the surface of GC TILs; inhibiting TIM-3 can improve T cell effector function and restore mitochondrial fusion [164]. By blocking mitochondrial biogenesis, the HIF-1α target gene vascular endothelial growth factor A (VEGF-A), which is highly expressed in GC, increases CD8^+^ T cell PD-1 expression; anti-VEGF-A plus anti-PD-1 therapy can restore T cell mitochondrial function and significantly increase anti-tumor efficacy [165]. Targeting mitochondrial metabolism to modulate immune checkpoint expression may provide a potential strategy for combining immune checkpoint inhibitors with GC therapy.

#### 3.6.5. Tumor Immune Evasion Involves GC Cells Hijacking Immune Cells’ Mitochondria

Research has shown that cancer cells can use tunneling nanotubes to “hijack” healthy mitochondria from immune cells, depriving TILs of metabolic substrates [166,167]. A more subtle reverse escape strategy was discovered by Hideki Ikeda and associates: cancer cells actively transfer their own mitochondria with “toxic” mtDNA mutations to TILs via nanotubes in addition to stealing healthy mitochondria. More significantly, the autophagy-inhibiting molecules (such as USP30) carried by these damaged mitochondria from the tumor surface enable them to withstand ROS-induced autophagic clearance within TILs. In the end, this results in homoplasmic replacement in T cells, which totally eliminates their ability to differentiate into effector memories and causes serious metabolic problems and cellular senescence [168]. According to research by Jaromir Novak and colleagues, deletion of Miro1, a crucial adaptor protein for mitochondrial transport along microtubules, can prevent mitochondrial horizontal transfer and slow the growth of tumors in melanoma models [169]. Interventions aimed at GC mitochondrial hijacking are still in their infancy. Targeted delivery of inhibitors to GC tissues is anticipated to develop into an entirely new approach to immunotherapy for GC in the future.

### 3.7. The Features of Cancer Stem Cells (CSCs) Are Shaped by Mitochondrial Dysfunction

As a crucial element of cellular phenotypic modification in the cascade model, mitochondrial metabolic reprogramming and disturbed oxidative stress homeostasis form the fundamental energy and signaling basis for the maintenance of CSC stemness [170,171]. Like normal cells, tumor stem cells’ aberrant mitochondrial structural morphology, mtDNA, oxidative phosphorylation, and autophagy not only control their stemness, proliferation, and apoptosis, but they also play a significant role in the failure of anticancer therapies [172]. Younghoon Kim and colleagues discovered that SEM subtype GCSCs in GC showed mitochondrial complex II (CII) dysfunction, which was characterized by decreased oxygen consumption rate, ROS accumulation, and apoptosis induction; in organoid and mouse models, α-D-tocopherol derivatives 10 and 17 targeting SDHC could selectively inhibit the proliferation of this subtype, demonstrating high efficacy and low toxicity [173]. Mitochondria-related lncRNA AC129507.1 is highly expressed in GC, and its knockdown can cause oxidative stress, ferroptosis, and disruptions in glucose and lipid metabolism. It also significantly inhibits cell migration and proliferation, indicating that it contributes to the malignant phenotype of GCSCs by controlling mitochondrial function [174]. Further demonstrating that an imbalance in mitochondrial homeostasis drives the evolution of GCSCs, LEMT2 overexpression sustains mitochondrial homeostasis and stimulates glycolysis by activating the mTOR pathway, significantly increasing tumor proliferation and metastasis [175]. Furthermore, via the p52-ZER6/IGF1R axis and TGFβ, DAZAP1, and clusterin-related molecular cascades, mitophagy also promotes GC stemness [176,177,178]. In conclusion, the primary cause of GCSCs’ drug resistance and development is an imbalance in mitochondrial homeostasis, which also directly controls the stemness and malignant phenotype of these cells.

### 3.8. Ferroptosis Is Regulated by Mitochondria

**Figure 2:**
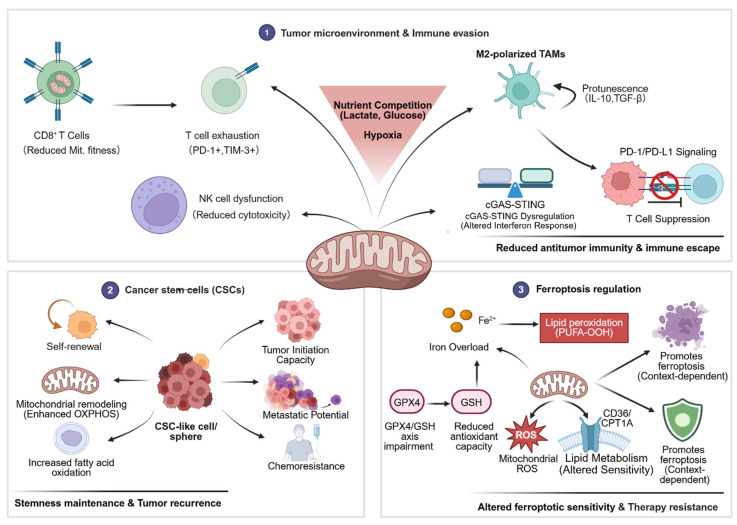
Roles of mitochondrial dysfunction in immune evasion, cancer stemness, and ferroptosis regulation in gastric cancer. Mitochondrial dysfunction contributes to gastric cancer progression by reshaping the TME, maintaining cancer stem cell (CSC) properties, and regulating ferroptosis sensitivity. In the TME, mitochondrial alterations promote T-cell exhaustion, NK-cell dysfunction, M2 macrophage polarization, cGAS–STING dysregulation, and PD-1/PD-L1-mediated immune suppression, leading to immune evasion. Mitochondrial metabolic remodeling further supports CSC self-renewal, tumor initiation, metastasis, and chemoresistance through enhanced oxidative phosphorylation and fatty acid oxidation. In addition, mitochondrial ROS production, iron overload, lipid peroxidation, and GPX4/GSH axis impairment alter ferroptosis sensitivity and contribute to therapy resistance in gastric cancer. Arrows indicate the direction of mitochondrial dysfunction-mediated regulation of immune cells, CSC-like properties, and ferroptosis-related processes. T-shaped lines denote inhibition.

In addition to being the main locations of ROS production in cells, mitochondria are also essential hubs for iron ion and lipid metabolism. They act as a central node that combines signals from upstream genomic damage, dynamic abnormalities, and metabolic disruptions, ultimately dictating the outcome of cell ferroptosis. With key features that set it apart from more conventional death mechanisms like apoptosis, necrosis, and autophagy, ferroptosis is a novel type of controlled cell death brought on by iron dependence and the buildup of lipid peroxides [179]. By controlling the TCA cycle, OXPHOS, iron/lipid metabolism, and ROS homeostasis, mitochondria directly control the ferroptosis threshold of GC cells [180]. Targeted silencing of SOX13 can reverse this phenotype and significantly increase the killing effect of ferroptosis inducers. Yang Hui and colleagues’ research revealed that SOX13 can transcriptionally activate SCAF1, promoting the assembly of mitochondrial respiratory chain supercomplexes and OXPHOS homeostasis, reducing mtROS overflow and lipid peroxidation, and mediating ferroptosis resistance in GC cells [181]. By preserving mitochondrial NAD/NADH homeostasis, blocking the ferroptosis driver prostaglandin-endoperoxide synthase 2, and lowering lipid peroxidation and mitochondrial membrane damage, mitochondrial-localized OXNAD1 overexpression mediates 5-fluorouracil resistance in GC. Resveratrol can directly bind to OXNAD1 to block its function, restoring ferroptosis sensitivity in resistant cells [182]. Lipid peroxidation is also linked to ETC complex I/III malfunction. In cancer cells with low GPX4 expression, pharmacological inhibition of mitochondrial respiratory chain complex I (MCI) can cause lipid peroxidation and ferroptosis; in cells with high GPX4 expression, this effect is somewhat mitigated [183]. The primary regulatory switches of ferroptosis in GC are the equilibrium of mitochondrial fission and fusion and mitophagy [184]. By stabilizing the mitochondrial inner membrane protease PARL, mitochondria-localized NPR1 inhibits mitophagy-dependent ferroptosis mediated by the PINK1-Parkin pathway, thereby mediating cisplatin resistance in GC, according to an in vitro experiment; silencing NPR1 can restore mitophagy flux and reverse the resistant phenotype [185]. One essential requirement for the start of ferroptosis is mitochondrial iron homeostasis. By downregulating the mRNA stability of the iron autophagy receptor NCOA4, the PLAGL2-STAU1 axis prevents ferritinophagy-mediated free iron release and mitochondrial iron absorption, shielding GC cells from ferroptosis. Peritoneal metastases of GC are closely linked to abnormalities in this route [186]. By controlling mitochondrial activity, the TME and pathogenic microorganisms in GC can also alter ferroptosis sensitivity. In GC, heterogeneous nuclear ribonucleoprotein C mediates oxaliplatin resistance and ferroptosis resistance, inhibits mitochondrial TCA cycle flow, decreases mtROS production, and increases lactate export and intracellular accumulation via upregulating MCT1 [187]. By activating the MEK/ERK/SRF pathway, upregulating the polyunsaturated ether lipid synthases AGPS/AGPAT3, and increasing the amount of polyunsaturated lipid substrates in the inner membrane of the mitochondria, CagA-positive *H. pylori* increases the ferroptosis susceptibility of GC cells [188] (Figure 2).

## 4. Mitochondria–Organelle Membrane Contact Network: Linking GC Development with Mitochondrial Dysfunction

**Figure 3:**
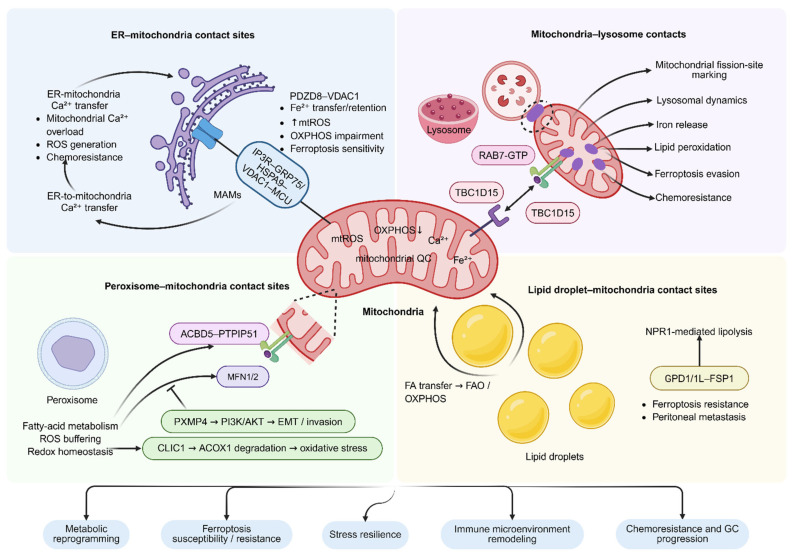
Mitochondria–Organelle Membrane Contact Network in GC. This schematic illustrates a mitochondria-centered inter-organelle communication network in gastric cancer (GC) cells. Mitochondria form dynamic membrane contact sites with the endoplasmic reticulum (ER), lysosomes, peroxisomes, and lipid droplets without membrane fusion. ER–mitochondria contact sites, especially mitochondria-associated ER membranes (MAMs), regulate Ca^2+^ transfer, Fe^2+^ homeostasis, ROS production, oxidative phosphorylation, ferroptosis sensitivity, and chemoresistance through axes such as IP3R–GRP75/HSPA9–VDAC1–MCU and PDZD8–VDAC1. Peroxisome–mitochondria contact sites (PoMCSs) participate in fatty-acid metabolism and redox regulation, whereas lipid droplet–mitochondria contact sites (LDMCSs) support fatty-acid transfer, mitochondrial β-oxidation, ferroptosis resistance, and metastatic adaptation. Mitochondria–lysosome contacts (MLCs) further coordinate mitochondrial quality control, iron handling, and therapy resistance. Together, these contact networks integrate calcium, iron, lipid, and redox signaling, thereby promoting metabolic reprogramming, stress adaptation, immune microenvironment remodeling, chemoresistance, and GC progression. Arrows indicate inter-organelle communication, ion or lipid transfer, and downstream functional effects. Dashed lines indicate membrane contact regions.

The idea that mitochondria are autonomous, functional organelles is being challenged by membrane contact sites (MCSs), which are areas of close proximity between organelles without membrane fusion. There is mounting evidence that mitochondria participate in a dynamic multi-organelle contact network with the endoplasmic reticulum (ER), lysosomes, peroxisomes, and lipid droplets [189]. By combining calcium homeostasis, lipid transport, redox regulation, and organelle QC, this network serves as a major signaling hub that modulates GC’s metabolic reprogramming, ferroptosis susceptibility, stress resilience, and immunological microenvironment remodeling [190] (Figure 3).

### 4.1. ER–Mitochondria Contact Sites in GC

ER–mitochondria contact sites, particularly mitochondria-associated ER membranes (MAMs), provide an important organelle-interaction perspective for understanding mitochondrial dysfunction in GC. Although direct evidence demonstrating structural remodeling of MAMs in GC remains limited, emerging studies on MAM-associated calcium transfer, mitochondrial iron homeostasis, ROS production, and chemoresistance suggest that ER–mitochondria contacts may contribute to GC progression and therapeutic resistance by reshaping mitochondrial functional states.

In GC, MAM-associated calcium signaling is closely linked to malignant phenotypes. High expression of the mitochondrial calcium uniporter MCU is associated with poor clinical outcomes and may influence mitochondrial function, energy metabolism, ROS generation, proliferation, apoptosis, and platinum resistance in GC cells [191]. This suggests that local ER-to-mitochondria Ca^2+^ transfer may be repurposed by GC cells to enhance metabolic adaptation, anti-apoptotic capacity, and chemoresistance. In addition, MUC20 variant 2 maintains mitochondrial calcium homeostasis and mitochondrial membrane potential in GC cells, thereby suppressing apoptosis and pyroptosis and increasing resistance to cisplatin and paclitaxel, further supporting the role of abnormal mitochondrial Ca^2+^ homeostasis in GC therapeutic resistance [192].

PDZD8 is a GC-relevant MAM-associated target. It is expressed in gastric adenocarcinoma tissues but is low or absent in normal gastric mucosa, intestinal metaplasia, and adenomas, and its expression correlates with invasion depth and pathological stage. Mechanistically, PDZD8 interacts with VDAC1 to regulate mitochondrial Fe^2+^ homeostasis. PDZD8 inhibition induces mitochondrial Fe^2+^ retention, increases mitochondrial ROS, and impairs oxidative phosphorylation, thereby suppressing GC cell proliferation. Moreover, combined sunitinib and pterostilbene treatment may inhibit PDZD8, promote mitochondrial iron accumulation and ROS production, enhance ferroptosis sensitivity, and exert stronger antitumor effects in xenograft models [193]. These findings suggest that PDZD8-related ER–mitochondria contacts support GC metabolic adaptation and may influence sensitivity to ferroptosis-inducing therapy.

GRP75/HSPA9 is an important bridging protein involved in ER–mitochondria Ca^2+^ transfer. In GC, GRP75 is upregulated in cisplatin-resistant cells. GRP75 knockdown disrupts the maintenance of mitochondrial membrane potential and suppresses NRF2, PI3K/AKT, HIF-1α, and c-Myc signaling, thereby weakening antioxidant, proliferative, and drug-resistant programs and restoring cisplatin sensitivity [194]. Although this study did not directly assess MAM structural alterations, the known role of GRP75 in the IP3R-GRP75-VDAC1 complex suggests that GC cells may maintain survival advantages under drug pressure by strengthening ER–mitochondria Ca^2+^/ROS coupling.

Overall, MAMs may be regarded as an important organelle-interaction platform linking mitochondrial dysfunction, metabolic adaptation, and therapeutic resistance in GC. Future studies using high-resolution imaging, quantitative MAM probes, GC organoids, and patient-derived models are needed to clarify the remodeling patterns of ER–mitochondria contacts across different GC molecular subtypes and to evaluate their therapeutic targetability.

### 4.2. Peroxisome–Mitochondria Contact Sites (PoMCSs) in GC

Basic studies have shown that the peroxisomal membrane protein ACBD5 can interact with the mitochondrial outer membrane protein PTPIP51 to form contact sites, thereby facilitating mitochondrial ROS transfer to peroxisomes and maintaining mitochondrial redox homeostasis [195]. In addition, mitochondrial fusion proteins such as MFN1 and MFN2 have also been implicated in regulating the spatial clustering and contact between peroxisomes and mitochondria [196]. Together, these findings suggest that PoMCSs may link lipid metabolism, ROS buffering, and mitochondrial functional maintenance. PXMP4, as a peroxisomal membrane protein, can promote EMT, migration, and invasion of GC cells through the PI3K/AKT signaling pathway [197], while CLIC1 enhances oxidative stress and malignant progression by promoting the ubiquitination-mediated degradation of ACOX1, a key enzyme in peroxisomal fatty acid β-oxidation [198].

### 4.3. Lipid Droplet–Mitochondria Contact Sites (LDMCSs) in GC

In nutrient-starved TMEs, fatty acids are transported by lipid droplets and mitochondria via MCSs, sustaining the energy source of cancer cells [199]. In GC, NPR1 can induce lipolysis of stored lipid droplets, release available fatty acids, and promote their entry into mitochondria to enhance oxidative phosphorylation, thereby driving GC metastasis [200]. In addition, lipid droplets may reduce lipid peroxidation and ferroptosis sensitivity during peritoneal dissemination. Oleic acid or adipocytes can induce lipid droplet accumulation in GC cells and decrease lipid peroxidation and cell death, whereas GPD1/1L-mediated lipid droplet formation upregulates FSP1, enhances ferroptosis resistance, and promotes peritoneal metastasis [201]. Although direct evidence of lipid droplet–mitochondria contact remodeling in GC remains limited, these findings suggest that lipid droplets may promote GC progression by regulating mitochondrial fatty acid utilization, OXPHOS, redox balance, and ferroptosis sensitivity.

### 4.4. Mitochondria–Lysosome Contacts (MLCs) in GC

MLC formation is associated with RAB7-GTP on the lysosomal membrane, whereas mitochondria-associated TBC1D15 promotes RAB7 GTP hydrolysis and participates in contact-site dissociation. Functionally, MLCs can mark mitochondrial fission sites and participate in the bidirectional regulation of mitochondrial networks and lysosomal dynamics [202]. Therefore, MLCs should not be simply equated with mitophagy, but rather regarded as dynamic contact platforms that regulate mitochondrial dynamics and lysosomal function. Chemoresistance and ferroptosis evasion in malignancies are tightly linked to MLC dysregulation. Although the intrinsic mitochondrial antioxidant system can lessen this effect, increased lysosome-mediated iron release can worsen mitochondrial lipid peroxidation. Ferroptosis is dependent on how these two elements are balanced [203]. It is important to note that research on MLCs in malignant tumors is currently restricted to melanoma, pancreatic cancer, and oral cancer; further systematic investigation is required to fully understand their unique roles, regulatory networks, and pathological importance in GC [204,205,206] (Table 1).

## 5. Important Signaling Pathways in GC Related to Mitochondria

**Figure 4:**
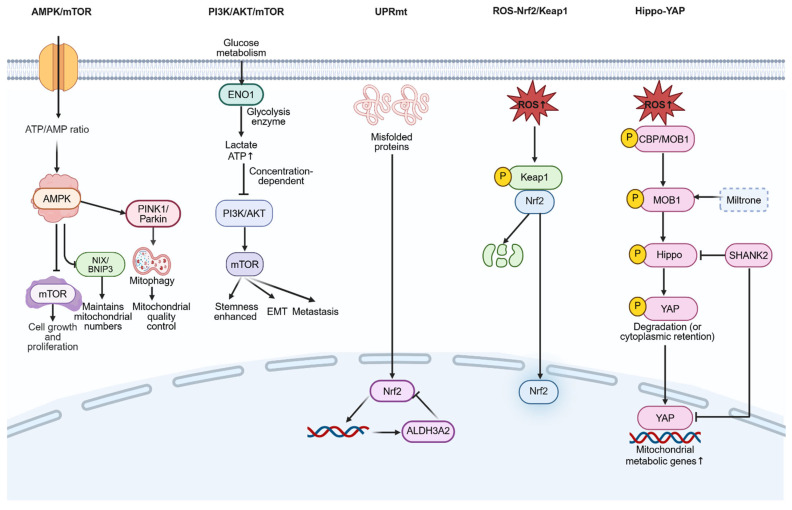
Important signaling pathways in GC related to mitochondria. Multiple signaling axes converge to promote gastric cancer progression. The AMPK/mTOR pathway regulates mitophagy and mitochondrial maintenance, while the ENO1-PI3K-AKT-mTOR loop drives EMT and metastasis. Mitochondrial stress (UPRmt and ROS) activates Nrf2-mediated antioxidant defenses. Concurrently, the Hippo-YAP axis integrates oxidative stress signals to modulate mitochondrial metabolic gene transcription. These synergistic networks facilitate metabolic adaptation and stress tolerance, ultimately enhancing GC cell survival and chemoresistance. Arrows indicate activation, signal transduction, metabolic flux, or downstream regulatory effects; blunt-ended lines indicate inhibition. The upward arrow indicates increased expression or activity. “P” denotes phosphorylation. Different colors are used to distinguish signaling molecules, mitochondrial-related regulators, transcription factors, metabolites, ROS, and pathway modules for visual clarity, rather than indicating quantitative differences.

A fundamental characteristic of GC, a highly diverse malignant tumor, is aberrant regulation of several cellular signaling pathways, the most important of which are the AMPK/mTOR, PI3K/AKT/mTOR, mitochondrial unfolded protein response (UPRmt), ROS-Nrf2/Keap1, and Hippo-Yes-associated protein (Yap) pathways (Figure 4).

The “gatekeeper” of cellular energy balance is AMP-activated protein kinase (AMPK). It can detect the drop in the ATP/AMP ratio brought on by malfunctioning mitochondria directly. A key hub connecting mitochondrial energy metabolism with cell proliferation, autophagy, and survival is the signaling axis created by AMPK and its downstream mammalian target of rapamycin (mTOR) [207]. It is noteworthy that Longo and colleagues discovered that AMPK regulates distinct mitophagy pathways in a way that balances the quantity and quality of mitochondria. AMPK promotes PINK1/Parkin-dependent mitophagy to maintain mitochondrial quality and inhibits NIX/BNIP3-dependent mitophagy to maintain mitochondrial quantity [208]. In GC, BiYanna and colleagues discovered that VDAC1 derived from exosomes (EV) can upregulate AMPK phosphorylation in a dose-dependent manner, inhibit mTOR, induce autophagy and mitophagy, and promote paclitaxel resistance in GC cells. In vivo experiments have validated the drug resistance effect of this pathway [209]. Furthermore, quercetin improves OXPHOS dysfunction, reduces the proliferation of GC cells and patient-derived organoids, and controls mitochondrial oxidative respiration in GC cells via activating AMPK [210].

One of the most often aberrantly activated pathways in GC is PI3K/AKT/mTOR, which is involved in a number of malignant biological characteristics, including angiogenesis, drug resistance, apoptotic suppression, cell proliferation, survival, and metastasis [211]. The key glycolytic enzyme ENO1 is abnormally overexpressed in GC, according to recent studies. This can lead to the accumulation of lactate and ATP, creating a positive feedback loop of PI3K/AKT activation and AMPK/mTOR imbalance: elevated ATP directly activates PI3K/AKT, which inhibits AMPK and relieves its suppression of mTOR, driving enhanced GC cell stemness, EMT, and metformin’s synergistic killing effect on GC [94]. By blocking this pathway, lowering mitochondrial membrane potential, encouraging cytochrome c release, and triggering the caspase cascade, phytochemicals like baicalin and isorhamnetin mediate mitochondrial apoptosis [212,213]. These discoveries offer new avenues for the treatment of GC with natural medications, but they are currently mostly restricted to in vitro studies, and in vivo metabolic stability and toxicity still need to be assessed.

A particular stress pathway called the mitochondrial unfolded protein response (UPRmt) is how mitochondria react to misfolded or aggregated proteins in the matrix. By increasing the expression of molecular chaperones and proteases via mitochondria-to-nucleus retrograde communication, it primarily restores mitochondrial protein homeostasis [214]. Overexpression of ALDH3A2 has been shown by Yuanyuan Ren and colleagues to block Nrf2 nuclear translocation, inhibit the expression of important UPRmt molecules lon protease 1 and heat shock protein 60, cause mitochondrial proteostasis collapse and dysfunction, and promote ferroptosis in GC cells. In vivo experiments have confirmed that it can significantly inhibit the growth of GC xenografts [12].

Under physiological conditions, Keap1 functions as the substrate recognition protein of the E3 ubiquitin ligase complex, mediating the ubiquitination and degradation of Nrf2; ROS derived from mitochondria can interfere with the Keap1-Nrf2 interaction and activate Nrf2, starting the transcription of target genes driven by antioxidant response elements (AREs), such as GPX4, solute carrier family 7 member 11, and heme oxygenase 1, thereby restoring cellular redox homeostasis [215,216]. One of the main ways that GC tumor cells withstand mitochondrial oxidative stress and fend against ferroptosis and chemotherapeutic medications is by the persistent aberrant activation of this pathway. Transmembrane protein 160 (TMEM160) is markedly overexpressed in GC tissues and cell lines, according to recent research. In order to promote K48-linked ubiquitination and degradation of Keap1, TMEM160 can enlist the E3 ubiquitin ligase tripartite motif containing 37. This continuously activates Nrf2, which transcriptionally upregulates GPX4 and solute carrier family 7 member 11, inhibits mitochondrial lipid peroxidation and ferroptosis, and eventually causes GC cells to become chemoresistant to cisplatin. Targeted TMEM160 knockdown can markedly improve GC cells’ ferroptosis sensitivity and chemotherapeutic responsiveness, according to in vivo studies. A new target for reversing GC medication resistance is provided by this study [217]. Furthermore, via activating the p62/Keap1/Nrf2 pathway, GDF15, a hallmark of poor prognosis in GC, can prevent ferroptosis, decrease mitochondrial ROS generation and lipid peroxidation, and increase GC cell proliferation and metastasis. A particular Nrf2 inhibitor can reverse this effect [218].

Through ROS-dependent cascade processes, mitochondrial dysfunction can directly control the Hippo pathway’s activation state, which is essential for controlling cell survival [219]. According to research by Wang Chuhang and colleagues, Tanshinone IIA, the active ingredient in Salvia miltiorrhiza, can cause mitochondrial dysfunction, autophagy damage, and ROS buildup in addition to drastically inhibiting GC cell growth and inducing apoptosis both in vivo and in vitro. It promotes MOB1 phosphorylation, which in turn triggers the Hippo pathway, resulting in YAP phosphorylation and degradation, via activating the CBP/MOB1 axis in a ROS-dependent manner. Its anti-tumor actions can be reversed by blocking the Hippo pathway, indicating that Tanshinone IIA’s primary strategy against GC is the activation of this system [220]. Although the in vivo pharmacokinetics and clinical translational potential of Tanshinone IIA require further investigation, these findings provide additional insights into the antitumor mechanisms of natural compounds in GC. Analysis of clinical samples reveals that SHANK2 is often overexpressed and amplified in GC. It can prevent YAP phosphorylation and degradation and encourage its nuclear translocation as an endogenous Hippo pathway inhibitor. It can also upregulate genes linked to mitochondrial metabolism, alter respiratory function, and aid GC cells in surviving nutritional stress. In GC patients, high SHANK2 expression is likewise strongly linked to a poor prognosis [221].

Through multi-node interactive crosstalk, the aforementioned mitochondrial-related signaling pathways constitute a complex regulatory network that collectively determines the destiny of GC cells rather than acting separately. For instance, via controlling ULK1, AMPK/mTOR directly influences PINK1/Parkin-mediated mitophagy [208]; the Nrf2/Keap1 and Hippo-YAP pathways can be simultaneously activated by mitochondrial ROS, working in concert to control oxidative stress and proliferation [216,219]. Additionally, UPRmt and mitophagy work in concert to regulate mitochondrial protein and structural homeostasis [222].

## 6. The Possibility of Using Mitochondrial Dysfunction as a GC Treatment Target

The primary treatment for GC, particularly in individuals with advanced stages, is chemotherapy. However, there are still three main obstacles to GC chemotherapy: primary and acquired resistance, as well as poor treatment response, severely limit efficacy; non-specific damage of chemotherapy drugs to normal cells results in multi-system adverse reactions; and the efficacy of single drugs is limited, and clinical practice heavily depends on combination therapy [223]. The occurrence, development, metastasis, and therapeutic resistance of GC are all regulated by mitochondria, which are now a major target for anti-tumor therapy. Currently, the great majority of potential approaches are still in the preclinical validation phase, with only cell lines and xenograft models demonstrating their effectiveness and mechanisms. Because of their proven safety in humans, repurposing tactics based on licensed medications like atovaquone, indomethacin, tipranavir, and zanamivir have a shorter translational pathway. On the other hand, low oral bioavailability and in vivo metabolic instability typically limit natural product-based candidate medications, requiring additional modification through nanodelivery technologies. The following text gathers typical candidate medications together with their validation progress, major limitations, and specific mechanisms of action in order to methodically outline the research stages and translational potential of diverse techniques.

### 6.1. Focusing on the Metabolic Reprogramming of Mitochondria

Mitochondria/STAT3-targetednanoplatform (ATO/CRNPs): Mitochondrial electron transport chain (ETC) complexes are core components of OXPHOS and serve as critical targets for metabolism-directed therapy. A self-assembling nanoplatform targeting ETC complex III (ATO/CRNPs) is formed by the self-assembly of atovaquone (ATO, an ETC complex III inhibitor) and the near-infrared photothermal agent CR. ATO inhibits OXPHOS by blocking electron transfer in ETC complex III, thereby reducing intracellular ATP levels and downregulating HSP70 expression, while also suppressing the STAT3 signaling pathway to synergistically enhance the photothermal therapeutic effect. This strategy achieved complete tumor eradication in a GC orthotopic transplantation tumor model without obvious systemic toxicity [224]. Notably, ATO’s pharmacokinetic properties and human safety have been well proven as an FDA-approved antimalarial medication. While GC’s indications are still in the preclinical stage, its anticancer research has reached clinical trials for non-small-cell lung cancer and acute myeloid leukemia. ATO/CRNP nanomedicine development offers a pharmacological basis for later clinical translation [225,226].

Quercetin: The LixianDing team discovered that quercetin targets the mitochondria-related transporter protein SLC1A5, inhibits the NRF2/GPX4 axis, and activates the p-Camk2/p-DRP1 pathway to increase mitochondrial fission and lipid peroxidation, ultimately causing ferroptosis in GC cells. Quercetin can raise iron buildup and lower GSH levels in GC cells. Ferroptosis inhibitor Fer-1 can reverse its anticancer impact, and SLC1A5 overexpression can considerably reduce this effect, offering experimental support for natural drug-targeted mitochondrial treatment for GC [227]. The in vivo metabolic stability, toxicity, and injection schedule of quercetin still require improvement, and this research is still restricted to in vitro cells and animal models.

Saikosaponin D (SSD): SSD is a naturally occurring triterpenoid saponin that possesses anti-tumor properties in a variety of malignancies. It is isolated from the traditional Chinese medicine Bupleurum. Experiments conducted both in vitro and in vivo have demonstrated that SSD can suppress the growth of transplanted tumors and inhibit GC cell viability, proliferation, migration, and invasion in a dose-dependent manner. It also upregulates cleaved caspase-3/9 to promote apoptosis and decreases global lactylation levels and Ki-67 expression. Mechanistically, SSD reduces global lactylation and H3 histone lactylation levels in GC cells by downregulating PKM2 expression, which limits glycolysis [228].

### 6.2. Focusing on Mitochondrial Autophagy

Indomethacin: The first anti-GC medication that has been shown to concurrently target autophagy and mitophagy is indomethacin. It can increase sensitivity to oxaliplatin chemotherapy, cause lysosomal malfunction to limit autophagic flux, and cause GC cells to undergo apoptosis without the need for mTOR [229]. When used in conjunction with chemotherapeutic medications, the TRPM2 inhibitor clotrimazole can function via the same mechanism and has a notable synergistic killing effect [230]. Further research revealed that indomethacin can directly target the mitochondrial deacetylase SIRT3, inhibit its deacetylase activity, prevent overactivation of mitophagy, cause mitochondrial oxidative stress imbalance and mtDNA damage, and ultimately cause mitochondria-dependent apoptosis in GC cells [231]. However, indomethacin’s mitochondrial-targeted anti-GC mechanism has only been confirmed in preclinical research, and the majority of its current clinical trials concentrate on non-tumor purposes. Furthermore, its usage as a single drug is limited by gastrointestinal side effects at high doses, which makes it more appropriate for development as a component of a combination therapy to sensitize chemotherapy [232].

8-paradol: Ginger’s phenolic chemical 8-paradol can increase mitophagy through the PINK1-Parkin pathway, causing GC cells to undergo apoptosis; in contrast, chloroquine suppresses mitophagy and can considerably reduce the effects of 8-paradol on mitochondrial dysfunction and cell death. This discovery makes clear how important mitophagy is to 8-paradol’s anticancer action [233].

WSGC@FA@PEG/PEI-SPIONs nano drug delivery system: The WSGC@FA@PEG/PEI-SPIONs nano-drug delivery system, according to research by Song Dongjian and colleagues, can target WSGC peptide delivery through folate receptors, specifically inhibit Notch pathway activity, thereby downregulating the expression of mitophagy-related proteins like PINK1 and Parkin, reverse oxaliplatin resistance in gastric adenocarcinoma cells, achieve 72% tumor growth in a nude mouse xenograft model, and simultaneously have MRI imaging capability, enabling accurate integrated diagnosis and treatment of GC [234].

Short-term fasting or dietary restriction: Short-term fasting, dietary restriction, or ATP-citrate lyase (ACLY) inhibition can lower cytosolic AcCoA levels, release NLRX1 from its autoinhibitory state, and trigger NLRX1-dependent mitophagy via interaction between its NACHT domain and LC3. However, by downregulating the ACLY-AcCoA axis, KRAS inhibitors can trigger this autophagy mechanism, making GC cells resistant to KRAS inhibitors. The tumor-suppressive effect of KRAS inhibitors can be increased by 60% when combined with the mitophagy inhibitor Mdivi-1 [235].

### 6.3. Focusing on the Dynamics of Mitochondria

Mdivi-1: By controlling mitochondrial dynamics and cellular oxygen consumption, the DRP1 inhibitor Mdivi-1 can prevent the growth of tumor cells [236]. It has potential anti-cancer actions and can stop the growth of malignant tumor cells, including lung and colon cancer, in in vitro trials [42]. In hypoxic GC, HIF-1α causes mitochondrial fission via the METTL3/IGF2BP3-DRP1 axis, which works in concert with lactate dehydrogenase A-mediated enhanced glycolysis to produce a significant amount of mtROS and activate NLRP3 inflammasome-mediated pyroptosis. This process can be effectively reversed by Mdivi-1 [47]. Although Mdivi-1 has demonstrated effectiveness in animal models, human validation (safety, specificity, and long-term application effects) has not yet been carried out.

SanggenonC: The root bark of the white mulberry, a traditional Chinese medicine having anti-inflammatory, antioxidant, and anticancer pharmacological properties, contains a flavonoid molecule called sanggenon C. According to studies, Sanggenon C can cause cell cycle arrest at the G0-G1 phase, impede the growth and colony formation of human GC cells, and encourage the cells’ apoptosis. Sanggenon C inhibits mitochondrial fission by blocking the extracellular signal-regulated kinase (ERK) signaling pathway, which mechanistically causes apoptosis. In vivo studies have demonstrated that Sanggenon C can inhibit the growth of xenograft tumors in nude mice without clearly harming major animal organs [48].

### 6.4. Focusing on the Antioxidant System in the Mitochondria

Propyl isothiocyanate (PITC): By lowering intracellular GSH levels in conjunction with GSH, PITC can lower mitochondrial antioxidant capacity, cause massive accumulation of mitochondrial ROS and DNA damage, trigger mitochondrial-dependent apoptosis and the p53 signaling pathway, and significantly inhibit the proliferation of both differentiated and undifferentiated GC cells [237].

Celastrol: Celastrol has the ability to directly bind to and inhibit the activity of peroxiredoxin 2 (Prdx2) in GC cells. This can result in a marked increase in intracellular ROS, which in turn causes ER stress and mitochondrial dysfunction, ultimately causing GC cells to undergo apoptosis via the mitochondrial pathway. It suppresses the growth of GC by reducing Prdx2 and increasing ROS generation, according to both in vitro and in vivo experiments (SGC-7901 and BGC-823 cells, as well as the SGC-7901 nude mouse xenograft model) [238].

Miltirone: Miltirone can directly bind to the Cys106 site of the mitochondrial antioxidant protein DJ-1, inhibiting its ROS scavenging function and causing a significant accumulation of mitochondrial ROS and a decrease in membrane potential, resulting in mitochondrial dysfunction. At safe doses, it also activates the Hippo signaling pathway, promoting YAP phosphorylation and degradation, which significantly inhibits the proliferation of GC cells both in vivo and in vitro, with no discernible systemic toxicity. This work is the first to identify DJ-1 as a novel therapeutic target for GC mitochondrial redox homeostasis [220].

Resveratrol: Red grapes naturally contain resveratrol, which has been demonstrated to cause apoptosis and prevent GC cells from proliferating, migrating, and invading. According to a study by Zhao Xuan and colleagues, resveratrol can directly target USP36, inhibit its deubiquitinase activity, promote the ubiquitin-mediated degradation of SOD2, disrupt mitochondrial redox homeostasis, and simultaneously initiate autophagy and ferroptosis processes. This results in a significant suppression of GC cell proliferation and metastasis, offering a new mechanistic basis for the use of natural medications that target the mitochondrial antioxidant system [239]. Additionally, a number of natural substances, such as ginsenoside F(2), shikonin, steroidal alkaloids, thymol, and wogonoside, can target the mitochondrial antioxidant system to have anti-GC effects. Their primary method is to control mitochondrial activity by focusing on ROS levels [213].

Zanamivir: The transcription factor SOX13 can cause mitochondrial metabolic reprogramming and increased antioxidant capacity, disrupt the assembly of mitochondrial respiratory chain supercomplexes, transcriptionally repress the mitochondrial structural regulator SCAF1, and mediate chemotherapy and ferroptosis resistance in GC cells. By directly targeting SOX13, promoting its ubiquitin-mediated degradation, restoring mitochondrial redox homeostasis and ferroptosis sensitivity, and increasing the effectiveness of chemotherapy, the anti-influenza medication Zanamivir offers a novel approach to repurposing outdated medications to treat resistant GC [181]. The FDA has approved zanamivir, an anti-influenza medication with proven human safety [240]. Its anti-GC activity targeting SOX13 has only been validated in preclinical models and requires further exploratory clinical studies.

### 6.5. Focusing on the Intrinsic Apoptotic Mechanism of Mitochondria

Mitochondria-targeting peptide Mito-FF: Mito-FF can selectively accumulate in the mitochondria of GC cells, disrupt membrane structure and encourage content leakage, directly activating the mitochondrial apoptosis pathway, and increase 5-FU chemotherapy sensitivity, demonstrating excellent anti-tumor activity in both in vitro and in vivo models, according to research by Kim Dong Jin and colleagues [241].

MoracinD: The natural small-molecule compound Moracin D has been shown in recent studies to directly target the BH3-binding groove of the Bcl-2 protein, disrupt its interaction with the pro-apoptotic protein Bax, promote Bax mitochondrial translocation and oligomerization, induce mPTP opening, which releases cytochrome c, activate the caspase-9/caspase-3 cascade, and ultimately trigger mitochondria-dependent apoptosis. Moracin D caused G2/M phase cell cycle arrest and decreased the growth of several GC cell lines in a dose-dependent manner, according to in vitro tests. Moracin D alone markedly reduced the growth of GC tumors in in vivo xenograft tumor models without clearly causing systemic harm. More significantly, by increasing the activation of the mitochondrial apoptotic pathway, Moracin D had a strong synergistic impact with 5-FU, completely reversing 5-FU chemoresistance and offering a unique combinational approach for the treatment of drug-resistant GC [242].

Uridine monophosphate (UMP): According to research by Cai Guodi and associates, UMP can stimulate the nuclear receptor NR4A1’s mitochondrial translocation, improve its direct interaction with Bcl-2, change Bcl-2’s anti-apoptotic function to pro-apoptotic activity, initiate the mitochondrial apoptosis pathway, and markedly reduce the growth of GC cells in vitro and in vivo. By increasing pyrimidine nucleotide stress, dihydroorotate dehydrogenase (DHODH) inhibitors can enhance the pro-apoptotic action of the UMP-NR4A1-Bcl-2 axis, offering a novel route for combination therapy [243].

Tipranavir: The LiFu team discovered that tipranavir, a medication that targets PRSS23, is capable of killing GC cell lines and GCSCs. Tipranavir (25 mg·kg^−1^·d^−1^, administered intraperitoneally for 8 days in succession) can markedly slow the growth of xenografts produced from GCSCs without causing clear harm. Mechanistically, it causes GCSC death by suppressing PRSS23 expression, releasing MKK3 from the PRSS23/MKK3 complex to activate p38 mitogen-activated protein kinase, and triggering the interleukin 24-mediated Bax/Bak mitochondrial apoptosis pathway. Additionally, it can eradicate drug-resistant and other cancer cell lines [244]. Tipranavir is a well-established anti-HIV protease inhibitor with FDA approval. However, its repositioning for GC-targeted PRSS23 study has only been validated preclinically utilizing xenograft models generated from tumor stem cells [245].

Additionally, a number of chemotherapeutic medications and natural substances, including 5-FU, apigenin, baicalein, and alpinetin, have anti-GC effects through the mitochondrial apoptosis pathway by controlling the Bcl-2 family protein balance, lowering the potential of the mitochondrial membrane, encouraging the release of pro-apoptotic factors, and triggering the caspase cascade [213] (Table 2 and Table 3).

## 7. Final Thoughts and Prospects

**Figure 5:**
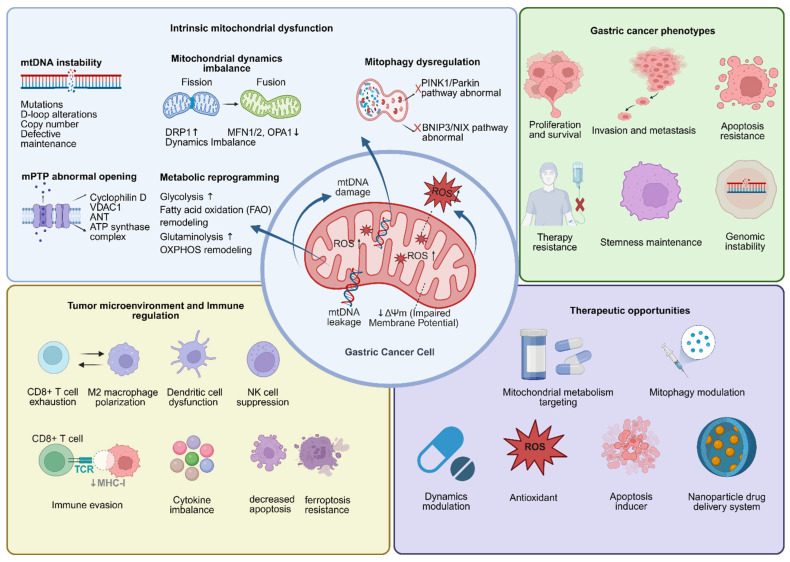
Overview of mitochondrial dysfunction in gastric cancer. Mitochondrial dysfunction in gastric cancer involves mtDNA instability, imbalance of mitochondrial dynamics, dysregulated mitophagy, abnormal mPTP opening, and metabolic reprogramming, leading to ROS accumulation, mtDNA damage, and impaired mitochondrial membrane potential. These alterations promote tumor progression, metastasis, stemness maintenance, immune evasion, and therapy resistance. Targeting mitochondrial metabolism, dynamics, mitophagy, oxidative stress, and apoptosis pathways represents promising therapeutic strategies for gastric cancer. Arrows indicate biological progression, signal transduction, metabolic flux, or downstream regulatory effects; blunt-ended lines indicate inhibition. Dashed arrows represent indirect or context-dependent regulation. Upward and downward arrows indicate increased or decreased expression/activity, respectively. Different colors are used to distinguish major mechanistic modules and do not indicate quantitative differences.

By highlighting important nodes like mitochondrial fission and fusion, mitophagy, antioxidant systems, intrinsic apoptotic pathways, and organelle interactions, this review methodically clarifies the multifaceted regulatory roles of mitochondrial abnormalities in GC and offers a number of translatable molecular targets for targeted interventions in GC. Mdivi-1, SanggenonC, Mito-FF and Moracin D are just a few of the mitochondrial-targeted small molecules, natural products, and nano-drug delivery systems that have been shown in numerous preclinical studies to effectively inhibit GC cell proliferation, induce mitochondria-dependent apoptosis, reverse drug resistance, and enhance chemotherapy sensitivity. These results show promising antitumor efficacy both in vitro and in vivo, along with possible safety. The translational potential of mitochondria-targeted therapy is further expanded by novel approaches such as ferroptosis induction, DHODH inhibition, mitochondrial apoptosis modulation, and integrated diagnostic and therapeutic nanoplatforms (Figure 5).

The core bottleneck restricting the clinical translation of mitochondrial-targeted therapy lies in the prominent mitochondrial heterogeneity across various molecular subtypes of GC. The four major GC subtypes classified by TCGA—CIN, MSI, EBV and GS—exhibit distinct mitochondrial metabolic and stress profiles [246]. The CIN subtype manifests robust metabolic activity and is highly dependent on mitochondrial oxidative metabolism and biogenesis [247]. MSI and EBV-positive subtypes display prominent immune phenotypes, where mitochondria-mediated immunometabolism regulates the TME and anti-tumor immunity [248]. The GS subtype mostly presents diffuse histological lesions accompanied by EMT and strong metabolic plasticity, which readily drives drug resistance and tumor progression [140]. Meanwhile, the molecular mechanisms underlying mitochondrial dysfunction in each subtype remain poorly elucidated, and subtype-specific targeted interventions are still lacking, with most relevant studies limited to preclinical investigations and preliminary clinical trials. Unique mitochondrial vulnerabilities intrinsic to each subtype render universal mitochondrial-targeted agents unable to achieve satisfactory anti-tumor efficacy, which severely hinders the development of individualized therapy for GC.

Second, the majority of medications that target mitochondria still do not have strict tumor selectivity, and their non-specific effects on mitochondria in healthy tissues may be harmful. Improving therapeutic specificity through prodrug activation, targeted administration, and microenvironment-responsive carriers is crucial [249]. Furthermore, the limited effectiveness of single-target techniques is frequently caused by the metabolic plasticity of GC cells, compensatory activation of antioxidant pathways, and adaptive resistance mediated by tumor stem cells. One important strategy for overcoming medication resistance is the sensible combination of immunotherapy, chemotherapy, and ferroptosis inducers [223]. Lastly, there are currently very few clinical-grade mitochondria-targeting medications with high selectivity and high druggability, and these medications’ pharmacokinetics, dosage schedules, and biomarker systems urgently require improvement [250].

In the future, the following directions may become key breakthroughs in promoting the clinical application of mitochondria-targeted strategies: integrating TCGA molecular subtyping, multi-omics data, single-cell sequencing results, and mitochondrial functional characteristics to establish a comprehensive evaluation model that reflects tumor metabolic status and therapeutic sensitivity; constructing mitochondrial feature scores (such as MitoScore) for prognostic assessment and precision medication prediction; developing mitochondria-targeted peptide conjugates, pH/hypoxia/enzyme-responsive nanomedicines, and antibody-drug conjugates to significantly enhance tumor accumulation and organelle targeting efficiency; establishing rational combination strategies of “mitochondria-targeted drugs with chemotherapy/immunotherapy/metabolic inhibitors” to systematically overcome resistance and enhance immunogenic cell death; and advancing novel therapeutic approaches, including mitochondrial transplantation, mitochondrial gene editing, copper death-targeted therapy, and mitochondria–immune–metabolic regulation, to provide new solutions for refractory and drug-resistant GC. Collectively, the integration of molecular stratification, mitochondrial precision targeting, and next-generation therapeutics may usher in a new era of personalized GC care, transforming current approaches to diagnosis, prognosis, and clinical management.

## Figures and Tables

**Table 1:** Major mechanisms of mitochondrial dysfunction and corresponding therapeutic targets in gastric cancer.

Mechanism of Mitochondrial Dysfunction	Representative Molecules/Pathways	Biological Consequences in GC	Potential Therapeutic Targets/Strategies	References
mtDNA instability	mtDNA mutations, mtDNA-CN, mtMSI, STING pathway	ROS accumulation, genomic instability, tumor progression	STING modulators, mtDNA damage repair strategies, DHODH inhibitors	[32,33,34,35,36]
Dysregulated mitochondrial dynamics	DRP1, MFN1/2, OPA1, FTO, ERK-DRP1 axis	Enhanced proliferation, invasion, metastasis, apoptosis resistance	DRP1 inhibitors, MFN2 restoration, mitochondrial fusion-promoting agents	[44,45,46,49,50,52]
Aberrant mitophagy	PINK1/Parkin, BNIP3, NIX, MUC1/ATAD3A axis	Tumor survival, stemness maintenance, chemotherapy resistance	Mitophagy inhibitors/modulators, BNIP3-targeted approaches, MUC1 inhibition	[63,64,67,68]
Abnormal mPTP regulation	CypD, VDAC1, MCU, MTCH2	Apoptosis evasion and drug resistance	CypD activators, HKII–VDAC disruptors, mPTP-opening agents	[73,74,75,76,77]
Glucose metabolic reprogramming	GLUT1/3, HK2, PKM2, ENO1	Enhanced glycolysis, tumor growth, immune suppression	Glycolysis inhibitors, HK2 inhibitors, PKM2-targeted therapy	[86,87,88,89,93,94]
Lipid metabolic reprogramming	CD36, CPT1A, ACAT1, circUBR5	Metastasis, ferroptosis resistance, immune evasion	CD36 inhibitors, FAO inhibitors, cholesterol metabolism-targeted therapy	[97,98,99,100]
Amino acid metabolic reprogramming	ASCT2, GLS, c-Myc, TGM2	Glutamine dependence, proliferation, immunosuppression	GLS inhibitors, ASCT2 inhibitors, glutamine restriction strategies	[103,106,107,108,109,110,111,112]
Immune microenvironment remodeling	cGAS-STING, PD-1/PD-L1, TIM-3, VEGF-A	T-cell exhaustion, NK-cell dysfunction, immune escape	Immune checkpoint blockade combined with mitochondrial-targeted therapy	[13,162,164,165]
Cancer stemness maintenance	OXPHOS, FAO, mitophagy-related pathways, LEMT2	Self-renewal, metastasis, therapeutic resistance	OXPHOS inhibitors, FAO inhibitors, CSC-targeted mitochondrial therapies	[173,174,175,176,177,178]
Ferroptosis resistance	GPX4, OXNAD1, NPR1, NCOA4, SOX13	Resistance to oxidative damage and chemotherapy	Ferroptosis inducers, GPX4 inhibition, mitochondrial ROS modulation	[181,183,186]
Mitochondria–organelle membrane contact network dysregulation	MAMs, PoMCSs, LDMCSs, MLCs; PDZD8–VDAC1, GRP75, ACBD5–PTPIP51	Remodels Ca^2+^ transfer, mitochondrial Fe^2+^,lipid transport, mitochondrial dynamics, ferroptosis sensitivity and chemoresistance in GC	Targeting MAM-associated Ca^2+^/iron/ROS signaling, lipid droplet–mitochondria fatty acid transfer, ferroptosis resistance pathways, and lysosome–mitochondria contact regulators	[189,190,191,192,193,194,195,196,197,198,199,200,201,202,203,204,205,206]

**Table 2:** Mitochondria-targeted preclinical therapeutic agents for gastric cancer.

Targeting Strategy	Agent/Intervention	Mechanism of Action	Experimental Model	References
Metabolic reprogramming	Quercetin	Targets SLC1A5; inhibits NRF2/GPX4;activates p-Camk2/p-DRP1; promotes mitochondrial fission and ferroptosis	In vitro and animal models	[227]
	Saikosaponin D	Downregulates PKM2; reduces global/H3 lactylation; inhibits glycolysis and induces apoptosis	In vitro and in vivo	[228]
Mitophagy	8-paradol	Activates PINK1/Parkin mitophagy; induces mitochondrial dysfunction via excessive mitophagy; triggers caspase-dependent mitochondrial apoptosis	In vitro and in vivo	[233]
	WSGC@FA@PEG/PEI-SPIONs	Folate receptor-targeted delivery; inhibits Notch pathway; downregulates PINK1/Parkin; reverses oxaliplatin resistance; MRI imaging	Nude mouse xenograft model	[234]
	Short-term fasting/ACLY inhibition	Reduces cytosolic AcCoA; releases NLRX1 autoinhibition; triggers NLRX1-dependent mitophagy; overcomes KRAS inhibitor resistance with Mdivi-1	Cell lines; KRAS inhibitor-resistant models	[235]
Mitochondrial dynamics	Mdivi-1	Drp1 inhibitor; blocks HIF-1α/METTL3/IGF2BP3-Drp1-mediated fission; reduces mtROS and NLRP3 pyroptosis	In vitro; hypoxic GC models	[47,236]
	Sanggenon C	Inhibits ERK; suppresses mitochondrial fission; induces G0-G1 arrest and apoptosis	In vitro; nude mouse xenograft model	[48]
Mitochondrial antioxidant system	PITC	Conjugates with GSH; reduces antioxidant capacity; promotes mtROS/DNA damage; activates p53-dependent apoptosis	Differentiated and undifferentiated GC cells	[238]
	Celastrol	Inhibits Prdx2; elevates ROS; induces ER stress and mitochondrial apoptosis	SGC-7901, BGC-823 cells; nude mouse xenograft model	[239]
	Miltirone	Covalently binds DJ-1 Cys106; impairs ROS scavenging; activates Hippo/YAP; inhibits proliferation	In vitro and in vivo	[220]
	Resveratrol	Targets USP36; promotes SOD2 ubiquitination; disrupts redox homeostasis; induces autophagy and ferroptosis	GC cell lines	[240]
	Others (ginsenoside F2, shikonin, etc.)	Modulate ROS and mitochondrial function	Various GC models	[213]
Intrinsic mitochondrial apoptosis	Mito-FF	Mitochondria-targeting peptide; disrupts membrane; activates apoptosis; sensitizes to 5-FU	In vitro and in vivo	[242]
	Moracin D	Binds Bcl-2 BH3 groove; promotes Bax translocation/oligomerization; activates caspase-9/-3; reverses 5-FU resistance	Multiple GC cell lines; xenograft model	[243]
	UMP	Induces NR4A1 mitochondrial translocation; converts Bcl-2 to pro-apoptotic function; synergizes with DHODH inhibitors	In vitro and in vivo	[244]
	Others (5-FU, apigenin, baicalein, etc.)	Regulate Bcl-2 family; decrease MMP; activate caspase cascade	_	[213]

**Table 3:** Mitochondria-targeted clinical therapeutic agents for gastric cancer.

Targeting Strategy	Medicine	Test Stage	Studied Model/Population	Dose	Result	Adverse Reaction	Identifier	References
Metabolic reprogramming	Atovaquone	Phase I trial (non-randomized, two-cohort window-of-opportunity translational clinical trial)	Patients with non-small-cell lung cancer	750 mg twice daily	the geometric mean HV of the treatment group was 55% lower than the control group (95%CI: 24% ~ 74%, *p* = 0.004)	The 750 mg/day dose was well-tolerated	NCT02628080 * (https://clinicaltrials.gov/study/NCT02628080)	[225]
Mitophagy	Indomethacin	Multi-center phase I trial	Patients with advanced metastatic solid tumors	Level 1: 25 mg three times daily Level 2: 50 mg three times daily Level 3: 75 mg three times daily	Combined indomethacin and CAPOX treatment is safe and reduces the concentrations of 12-S-HHT	Indomethacin-related adverse events were mostly mild Grade 1–2; only one Grade 3 event recorded, no Grade 4/5 toxicities.	NCT01719926 * (https://clinicaltrials.gov/study/NCT00820612)	[232]
Mitochondrial antioxidant system	Zanamivir	Phase II trial (Single-Arm Trial)	Patients with severe or progressive influenza	600 mg twice daily	85% of patients experienced any AE; Grade 3/4 AEs occurred in 44% of subjects	Liver injury (10%), rash (3%), thrombophlebitis/venous thrombosis (3%)	NCT01014988 * (https://clinicaltrials.gov/study/NCT01014988)	[241]
Intrinsic mitochondrial apoptosis	Tipranavir	Phase III trials (randomized, open-label comparative)	HIV-infected patients	Tipranavir 500 mg + ritonavir 200 mg twice daily	ITT treatment response rate: TPV/r 33.6% vs. CPI/r 15.3% (*p* < 0.001). Median time to treatment failure: TPV/r 113 days vs. CPI/r 0 days (*p* < 0.001). Only 26.1% of control patients remained on original regimen at week 48, versus 65.1% in TPV/r arm.	Diarrhea (11%), nausea (7%), pyrexia (4.6%), fatigue (4.0%), headache (3.1%), rash (2–14%)	—	[246]

Table note: The asterisk (*) indicates that the date of access for all URLs was 16 June 2026.

## Data Availability

No new data were created or analyzed in this study. Data sharing is not applicable to this article.

## Abbreviations

GC	Gastric Cancer
mtDNA	Mitochondrial DNA
mPTP	Mitochondrial Permeability Transition Pore
TME	Tumor Microenvironment
CSCs	Cancer Stem Cells
MSI	Microsatellite Instability
CIN	Chromosomal Instability
GS	Genomically Stable
EBV	Epstein–Barr Virus
TCGA	The Cancer Genome Atlas
QC	Quality Control
TCA	Tricarboxylic Acid Cycle
OXPHOS	Oxidative Phosphorylation
ATP	Adenosine Triphosphate
mtMSI	Mitochondrial Microsatellite Instability
mtDNA-CN	Mitochondrial DNA Copy Number
DRP1	Dynamin-Related Protein 1
PINK1	PTEN-Induced Kinase 1
PRKN	Parkin RBR E3 Ubiquitin Ligase
BNIP3	BCL2/Adenovirus E1B 19kDa Interacting Protein 3
NIX	BCL2/Adenovirus E1B 19kDa Interacting Protein 3 Like
BNIP3L	BCL2/Adenovirus E1B 19kDa Interacting Protein 3 Like
ANT	Adenine Nucleotide Translocase
GLUT	Glucose Transporter
HK2	Hexokinase 2
ENO1	Enolase 1
PKM2	Pyruvate Kinase M2
CD36	Cluster of Differentiation 36
CPT1A	Carnitine Palmitoyltransferase 1A
ACAT1	Acyl-Coenzyme A:Cholesterol Acyltransferase 1
CYP19A1	Cytochrome P450 Family 19 Subfamily A Member 1
ASCT2	Alanine-Serine-Cysteine Transporter 2
GLS	Glutaminase
GLS1	Glutaminase 1
TGM2	Transglutaminase 2
IDO1	Indoleamine 2,3-Dioxygenase 1
*H. pylori*	*Helicobacter pylori*
CagA	Cytotoxin-Associated Gene A
VacA	Vacuolating Cytotoxin A
HIF-1α	Hypoxia-Inducible Factor 1α
SIRT3	Sirtuin 3
YAP	Yes-Associated Protein
MAMs	Mitochondria-Associated Endoplasmic Reticulum Membranes
MCSs	Membrane Contact Sites
GRP75	Glucose-Regulated Protein 75
AMPK	AMP-Activated Protein Kinase
mTOR	Mammalian Target of Rapamycin
PI3K	Phosphatidylinositol 3-Kinase
UPRmt	Mitochondrial Unfolded Protein Response
Nrf2	Nuclear Factor Erythroid 2-Related Factor 2
Keap1	Kelch-Like ECH-Associated Protein 1
ARE	Antioxidant Response Element
GPX4	Glutathione Peroxidase 4
TMEM160	Transmembrane Protein 160
GDF15	Growth Differentiation Factor 15
MOB1	Mps One Binder Kinase Activator 1
SHANK2	SH3 and Multiple Ankyrin Repeat Domains 2
STAT3	Signal Transducer and Activator of Transcription 3
NF-κB	Nuclear Factor Kappa B
NFAT	Nuclear Factor of Activated T Cells
TOX	Thymocyte Selection-Associated High Mobility Group Box
NR4A	Nuclear Receptor Subfamily 4 Group A
PGC-1α	Peroxisome Proliferator-Activated Receptor Gamma Coactivator 1-Alpha
NK	Natural Killer
NLRP3	NOD-Like Receptor Family Pyrin Domain Containing 3
ox-mtDNA	Oxidatively Damaged Mitochondrial DNA
MDSCs	Myeloid-Derived Suppressor Cells
MHC-I	Major Histocompatibility Complex Class I
TIM-3	T-Cell Immunoglobulin and Mucin Domain-Containing 3
VEGF-A	Vascular Endothelial Growth Factor A
USP30	Ubiquitin-Specific Peptidase 30
Miro1	Mitochondrial Rho GTPase 1
SDHC	Succinate Dehydrogenase Complex C
SOX13	SRY-Box Transcription Factor 13
SCAF1	SR-Related CTD Associated Factor 1
OXNAD1	Oxidoreductase NAD Binding Domain Containing 1
NCOA4	Nuclear Receptor Coactivator 4
STAU1	Staufen Double-Stranded RNA Binding Protein 1
MCT1	Monocarboxylate Transporter 1
AGPS	Alkylglycerone Phosphate Synthase
FLCN	Folliculin
PIKFYVE	Phosphoinositide Kinase FYVE-Type Containing
PARL	Presenilin-Associated Rhomboid-Like
MARCH8	Membrane-Associated Ring-CH-Type Finger 8
NPR1	Natriuretic Peptide Receptor 1
LACTB	Beta-Lactamase Like
MUC1	Mucin 1
ATAD3A	ATPase Family AAA Domain Containing 3A
NOXO1	NADPH Oxidase Organizer 1
ROS	Reactive Oxygen Species
mtROS	Mitochondrial Reactive Oxygen Species
ETC	Electron Transport Chain
SSD	Saikosaponin D
PITC	Propyl Isothiocyanate
Prdx2	Peroxiredoxin 2
USP36	Ubiquitin-Specific Peptidase 36
SOD2	Superoxide Dismutase 2
Mito-FF	Mitochondria-Targeting Peptide FF
Bcl-2	B-Cell Lymphoma 2
Bax	BCL2-Associated X Protein
UMP	Uridine Monophosphate
NR4A1	Nuclear Receptor Subfamily 4 Group A Member 1
DHODH	Dihydroorotate Dehydrogenase
PRSS23	Protease Serine 23
MKK3	Mitogen-Activated Protein Kinase Kinase 3
SERCA	Sarco/Endoplasmic Reticulum Calcium ATPase
METTL3	Methyltransferase Like 3
IGF2BP3	Insulin Like Growth Factor 2 MRNA Binding Protein 3
ACLY	ATP-Citrate Lyase
NLRX1	NLR Family Member X1
Mdivi-1	Mitochondrial Division Inhibitor 1
